# Impact of oral preoperative and perioperative immunonutrition on postoperative infection and mortality in patients undergoing cancer surgery: systematic review and meta‐analysis with trial sequential analysis

**DOI:** 10.1002/bjs5.50314

**Published:** 2020-06-23

**Authors:** F. Buzquurz, R. D. Bojesen, C. Grube, M. T. Madsen, I. Gögenur

**Affiliations:** ^1^ Department of Surgery Slagelse Hospital Slagelse Denmark; ^2^ Centre for Surgical Science Zealand University Hospital Køge Denmark

## Abstract

**Background:**

Infectious complications occur in 4–22 per cent of patients undergoing surgical resection of malignant solid tumours. Improving the patient's immune system in relation to oncological surgery with immunonutrition may play an important role in reducing postoperative infections. A meta‐analysis was undertaken to evaluate the potential clinical benefits of immunonutrition on postoperative infections and 30‐day mortality in patients undergoing oncological surgery.

**Methods:**

PubMed, Embase and Cochrane Library databases were searched to identify eligible studies.

**Eligible studies had to include patients undergoing elective curative surgery for a solid malignant tumour and receiving** immunonutrition orally before surgery, including patients who continued immunonutrition into the postoperative period. The main outcome was overall infectious complications; secondary outcomes were surgical‐site infection (SSI) and 30‐day mortality, described by relative risk (RR) with trial sequential analysis (TSA). Risk of bias was assessed according to Cochrane methodology.

**Results:**

Some 22 RCTs with 2159 participants were eligible for meta‐analysis. Compared with the control group, immunonutrition reduced overall infectious complications (RR 0·58, 95 per cent c.i. 0·48 to 0·70; *I* ^2^ = 7 per cent; TSA‐adjusted 95 per cent c.i. 0·28 to 1·21) and SSI (RR 0·65, 95 per cent c.i. 0·50 to 0·85; *I* ^2^ = 0 per cent; TSA‐adjusted 95 per cent c.i. 0·21 to 2·04). Thirty‐day mortality was not altered by immunonutrition (RR 0·69, 0·33 to 1·40; *I* ^2^ = 0 per cent).

**Conclusion:**

Immunonutrition reduced overall infectious complications, even after controlling for random error, and also reduced SSI. The quality of evidence was moderate, and mortality was not affected by immunonutrition (low quality). Oral immunonutrition merits consideration as a means of reducing overall infectious complications after cancer surgery.

## Graphical abstract

A systematic review and meta‐analysis with trial sequential analysis was performed, summarizing studies on oral preoperative and perioperative immunonutrition in patients undergoing cancer surgery, and the impact on postoperative infection and mortality. Meta‐analysis found that immunonutrition reduced overall infectious complications and surgical‐site infection, even after controlling for random error.

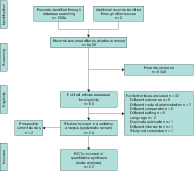

Reduces infection but not mortality

## Abstract (es)

**Antecedentes:**

Entre un 4‐22% de los pacientes a los que se realiza una resección quirúrgica de tumores sólidos malignos presentan complicaciones infecciosas. Mejorar el sistema inmunitario del paciente quirúrgico oncológico mediante inmunonutrición puede tener un papel relevante en la reducción de las infecciones postoperatorias. Se realizó un metaanálisis para evaluar los posibles beneficios clínicos de la inmunonutrición en las infecciones postoperatorias y la mortalidad a los 30 días en pacientes sometidos a cirugía oncológica.

**Métodos:**

Se realizó una búsqueda en las bases de datos de Pubmed, Embase y Cochrane para identificar los estudios clave. Se consideraron aquellos estudios que incluyeron pacientes con cirugía curativa electiva de un tumor maligno sólido que recibieron inmunonutrición por vía oral antes de la cirugía, así como también los que siguieron con inmunonutrición en el postoperatorio. La variable principal fueron las complicaciones infecciosas generales y las secundarias fueron la infección de la herida quirúrgica y la mortalidad a los 30 días, presentadas como el riesgo relativo (RR) obtenido a partir en un análisis secuencial de experimentos (*trial sequential analysis*, TSA). El riesgo de sesgo se evaluó según la metodología Cochrane.

**Resultados:**

Para el metaanálisis se identificaron 22 ensayos clínicos con 2.075 participantes. En comparación con el grupo de control, la inmunonutrición redujo las complicaciones infecciosas generales (RR 0,58, i.c. del 95% 0,48‐0,70, I^2^ = 7%, TSA ajustado i.c. del 95% 0,28‐1,21) y las infecciones de la herida quirúrgica (RR 0,65, i.c. del 95% 0,50‐0,85, I^2^ = 0%, TSA ajustado, i.c. del 95% 0,21‐2,04). No hubo diferencias en la mortalidad a los 30 días (RR 0,69, i.c. del 95% 0,32‐1,4, I^2^ = 0%).

**Conclusión:**

la inmunonutrición redujo las complicaciones infecciosas generales, incluso después de controlar el error aleatorio. La inmunonutrición también redujo la infección de la herida quirúrgica. La calidad de la evidencia fue moderada y la mortalidad no se vio afectada por la inmunonutrición (baja calidad). La inmunonutrición oral debería ser tenida en cuenta como una forma de reducir las complicaciones infecciosas generales después de la cirugía del cáncer.

## Introduction

Even with optimal patient care, minimally invasive surgery and enhanced recovery after surgery, infectious complications remain a problem. Postoperative infectious complications occur in 4–22 per cent of patients undergoing surgical resection for cancer^1^, ^2^, ^3^, ^4^. New interventions aiming at decreasing the risks of infectious complications by modulating the immune system have been proposed. One such intervention is immunonutrition. By providing key nutrients, immunonutrition may play an important role in reducing postoperative infections. The most studied nutrients in immunonutrition formulas are arginine, glutamine, omega‐3 fatty acids and nucleotides. Orally administered immunonutrition has several clinical advantages. as it is non‐invasive, self‐administrable, safe, and complies with enhanced recovery after surgery protocols.

The proinflammatory response is crucial after cancer surgery, but high levels of inflammation might promote immunosuppression. Immunonutrients may work by reducing the postoperative production of proinflammatory lipid mediators and cytokines, in addition to promoting lymphocyte production and function^5^.

The objective of this study was to evaluate the potential clinical benefits of immunonutrition given in relation to the timing of surgery on postoperative infections and 30‐day mortality in patients undergoing oncological surgery in comparison with patients not receiving immunonutrition.

## Methods

This review was performed in accordance with the guidelines outlined in the Cochrane Handbook^6^ and reported in line with the PRISMA statement^7^, and was registered prospectively on PROSPERO (CRD42019135854).

### Eligibility criteria

Studies of patients undergoing elective curative resection of a solid malignancy were included. Studies including patients under the age of 18 years or with stage IV cancer were excluded. Immunonutrition had to be administered within 30 days, and at the latest 5 days, before surgery. Continuation into the in‐hospital postoperative period was allowed, but only by the oral route or tube feeding. Immunonutrition was defined by having at least two of the following substances in the nutritional formulation: arginine, glutamine, omega‐3 fatty acids, RNA and nucleotides. Studies were included if control groups received placebo or standard of care with no immunonutrition before or after surgery.

The primary outcome was overall infectious complications and secondary outcomes were surgical‐site infection (SSI) and 30‐day mortality. Studies with at least one of the relevant outcomes were included. Only RCTs and prospective cohort studies were included.

### Information sources and search strategy

PubMed, Cochrane Library and Embase databases were searched, with no restrictions on publication date. Searches were restricted to human studies only. The last search was done in August 2019. Search strategies were developed in collaboration with a health science information specialist from Region Zealand's Reference Library (*Appendix* *S1*, supporting information).

### Study selection

Searches from the three databases were extracted and uploaded to Covidence (Veritas Health Innovation, Australia; www.covidence.org). The articles were screened independently by two reviewers in two stages; titles and abstracts were assessed for eligibility in the first stage and at full‐text level in the second stage. In case of any disagreement between reviewers, a third reviewer was consulted and consensus reached.

### Data collection process and items

Data were extracted independently by two reviewers using Excel® 2017 (Microsoft, Redmond, Washington, USA) into a predesigned data spreadsheet. Extracted data items were: nutritional state, measures used to assess nutritional state, anatomical site of cancer, country, name of first author, year, study design, number of patients, sex distribution, time point (preoperative or perioperative) and duration (days) of immunonutrition administration, experimental drug (nutrients, dose, brand name), treatment in control group, compliance, adverse effects and/or tolerance, and outcomes (overall infectious complications, SSI and 30‐day mortality).

### Risk of bias in individual studies and study quality assessment

Risk of bias was assessed independently by two reviewers. For RCTs, risk of bias was assessed in Review Manager version 5.3 (The Cochrane Collaboration, The Nordic Cochrane Centre, Copenhagen, Denmark) using the Cochrane Collaboration's tool^6^. Risk of bias for cohort studies was assessed according to the Newcastle–Ottawa system^8^. Publication bias was investigated using funnel plots and Egger's test^9^. The quality of the evidence was judged using Grading of Recommendations, Assessment, Development and Evaluation (GRADE). The aspects assessed in GRADE were study design, risk of bias, inconsistency, indirectness, imprecision and publication bias^10^, ^11^. Quality of evidence was rated as high, moderate, low or very low.

### Statistical analysis

#### Meta‐analysis

Meta‐analyses were performed in R statistical software version 3.5.2 (The R Foundation for Statistical Computing, Vienna, Austria). Meta‐analyses on dichotomous outcomes were performed using the random‐effects model, calculating risk ratios (RRs) with the Mantel–Haenszel method. The Hartung–Knapp–Sidik–Jonkman method of adjusting 95 per cent c.i. was applied to the random‐effects model^12^. The DerSimonian–Laird method was used as estimator for τ^2^, and a continuity correction of 0·5 was used for studies with zero cell frequencies according to Cochrane methodology^6^.

Prediction intervals supplying an estimate of the expected effect sizes on future studies were calculated^13^.

Heterogeneity was assessed by χ^2^ testing and *I* ^2^ statistics. When the *I* ^2^ value was less than 25 per cent, a fixed‐effect model was presented as a sensitivity analysis.

Preplanned subgroup analyses were: preoperative *versus* perioperative immunonutrition; active comparator (defined as receiving isocaloric or isocaloric + isonitrogenous supplement) *versus* no supplement in the control group; anatomical site of surgery; and nutritional state (well nourished *versus* malnourished participants). *Post hoc* analysis comparing blinded and unblinded studies was done for studies in which the control group received an active comparator.

#### Trial sequential analysis

Trial sequential analysis (TSA) was applied to address the risk of random error associated with sparse data and/or multiple testing^6^, ^14^, ^15^, ^16^, ^17^.

To prevent *post hoc* data‐driven testing, several criteria must be defined *a priori* to the TSA being run. The criteria consist of: proportion of events in the control group for a binary outcome (Pc); a realistic relative risk reduction (RRR) for a binary outcome between groups; α; β; and diversity (D^2^), estimated by the TSA model^18^, ^19^.

The proportion of events in control groups was based on the frequency shown in the present review (dichotomous outcomes). RRR values of 10 and 20 per cent were chosen pragmatically to represent a conservative and a liberal intervention effect respectively. An α level of 5 per cent was chosen for the primary outcome, and 3·6 per cent for secondary outcomes (two comparisons) in line with the guidelines described by Jakobsen and colleagues^18^. A β value of 10 per cent (power of 90 per cent) was chosen, and diversity (a measure of between‐study heterogeneity) was determined using the TSA model (http://www.ctu.dk/tsa/) (*Appendix* *S2*, supporting information).

## Results

**Figure 1:**
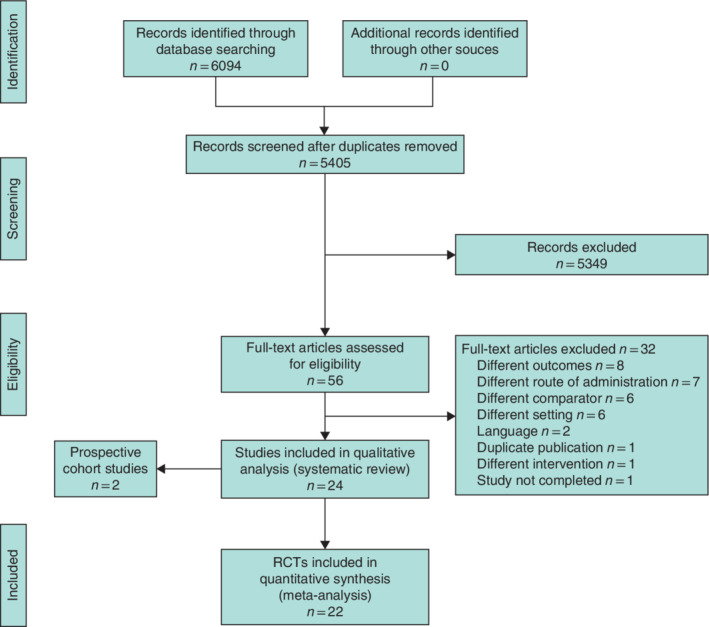
PRISMA diagram for the review

The searches resulted in 6094 articles; after removal of duplicates and screening of titles and abstracts, 56 were left for full‐text screening. At the full‐text screen, 32 articles were excluded for reasons listed in the PRISMA diagram (*Fig*. *1*). Twenty‐two RCTs^20^, ^21^, ^22^, ^23^, ^24^, ^25^, ^26^, ^27^, ^28^, ^29^, ^30^, ^31^, ^32^, ^33^, ^34^, ^35^, ^36^, ^37^, ^38^, ^39^, ^40^, ^41^ were available for meta‐analysis with 21 studies^20^, ^21^, ^22^, ^23^, ^24^, ^25^, ^26^, ^27^, ^28^, ^29^, ^30^, ^32^, ^33^, ^34^, ^35^, ^36^, ^37^, ^38^, ^39^, ^40^, ^41^ providing results on overall infectious complications, 17 studies^20^, ^21^, ^22^, ^24^, ^25^, ^26^, ^27^, ^30^, ^34^, ^36^, ^37^, ^38^, ^39^, ^41^ on SSI, and 13 studies^20^, ^21^, ^22^, ^25^, ^26^, ^27^, ^28^, ^30^, ^32^, ^33^, ^34^, ^36^, ^41^ on 30‐day mortality. Two prospective cohort studies^42^, ^43^ were included in the systemic review but not in the meta‐analysis (*Tables S1* and *S2*, supporting information). A list of the authors contacted is shown in *Table S3* (supporting information).

### Study characteristics

Characteristics of included studies are summarized in *Table* *1* and *Table S4* (supporting information). Twelve studies investigated perioperative immunonutrition, nine preoperative immunonutrition, and three had both perioperative and preoperative immunonutrition groups. In the studies investigating perioperative immunonutrition, postoperative immunonutrition was administered by tube feeding, except in two studies^28^, ^36^ where it was given orally.

**Table 1:** Details of studies eligible for meta‐analysis

				**Duration (days)**		**Immunonutrition dose**			
**Reference**	**Country**	**Sample** **size**	**Anatomical** **site**	**Preop.**	**Postop.**	**Preop.**	**Postop.**	**Nutrients** **in immunonutrition**	**Control group**
Braga *et al*.^20^	Italy	171	Mixed GI	7	7	1000 ml/day	Increased until 1500 ml/day	Arginine, omega‐3, RNA	Isocaloric supplement
Braga *et al*.^21^	Italy	150	Mixed GI	7	Until oral food consumption	1000 ml/day	Initially 10 ml/h, increased until 28 kcal per kg per day	Arginine, omega‐3, RNA	No supplement
				7	–	1000 ml/day	–		
Braga *et al*.^22^	Italy	200	Colorectal	5	n.s.	1000 ml/day	n.s.	Arginine, omega‐3, RNA	2 control groups: isocaloric supplement and no supplement
				5	–	1000 ml/day			
Campillo *et al*.^23^	Spain	84	Colorectal	8	–	700 ml/day	–	Arginine, omega‐3, RNA	No supplement
Felekis *et al*.^24^	Greece	40	Head/neck	5	8	Harris–Benedict	Harris–Benedict	Arginine, omega‐3, RNA	Isocaloric supplement
Fujitani *et al*.^25^	Japan	233	Gastric	5	–	1000 ml/day	–	Arginine, omega‐3, RNA	No supplement
Gade *et al*.^26^	Denmark	24	Pancreatic	7	–	250–1000 ml/day	–	Arginine, omega‐3, RNA	No supplement
Gianotti *et al*.^27^	Italy	305	Mixed GI	5	Until oral food consumption	1000 ml/day	1·5 litres/day	Arginine, omega‐3, RNA	No supplement
				5	–	1000 ml/day	–	Arginine, omega‐3, RNA	No supplement
Hamilton‐Reeves *et al*.^28^	USA	29	Bladder	5	5	n.s.	n.s.	Arginine, omega‐3, RNA	Supplement (360 and 200 kcal per carton for control and experimental solution respectively)
Hamza *et al*.^29^	UK	42	Pancreatic	14	7	600 ml/day	Initially 25 ml/h, increased until 25 kcal per kg per day	Arginine, omega‐3, RNA	Isocaloric supplement
Helminen *et al*.^30^	Finland	60	Mixed GI	5	5	900 ml/day	900 ml/day	Arginine, omega‐3, RNA	No supplement
Horie *et al*.^31^	Japan	67	Colorectal	5	–	750 ml/day	–	Arginine, omega‐3, RNA	No supplement
Hübner *et al*.^32^	Switzerland	123	Mixed GI	5	–	500–1000 ml/day	–	Arginine, omega‐3, RNA	Isocaloric + isonitrogenous supplement
Kanekiyo *et al*.^33^	Japan	40	Oesophageal	7	7	750 kcal/day	750 kcal/day	Arginine, omega‐3, RNA	Isocaloric supplement
McCarter *et al*.^34^	USA	24	Mixed GI	7	–	750 ml/day	–	Arginine, omega‐3	Isocaloric + isonitrogenous supplement
Mikagi *et al*.^35^	Japan	26	Liver	5	–	750 ml/day	–	Arginine, omega‐3, RNA	No supplement
Moya *et al*.^36^	Spain	244	Colorectal	7	5	400 ml/day	400 ml/day	Arginine, omega‐3, RNA	Supplement (125 and 151 calories per 100 ml for control and experimental solution respectively)
Okamoto *et al*.^37^	Japan	60	Gastric	7	–	750 ml/day	–	Arginine, omega‐3, RNA	Isocaloric supplement
Seguin *et al*.^38^	France	35	Liver	7	3	900 ml/day	n.s.	Arginine, omega‐3, RNA	Isocaloric supplement
Senkal *et al*.^39^	Germany	154	Upper GI	5	5	1000 ml/day	Increased from 20 to 80 ml/h	Arginine, omega‐3, RNA	Isocaloric supplement
Turnock *et al*.^40^	New Zealand	8	Head/neck	5	5	900 ml/day	Increased until 28 kcal per kg per day	Arginine, omega‐3, RNA	No supplement
Uno *et al*.^41^	Japan	40	Liver, gallbladder, bile duct	5	–	1000 kcal/day	–	Arginine, omega‐3, RNA	No supplement

GI, gastrointestinal; n.s., not stated.

In the majority of the studies, the duration of follow‐up was 30 days after surgery^26^, ^28^, ^32^, ^36^, ^38^, ^41^, 30 days after discharge^20^, ^21^, ^22^, ^23^, ^27^, ^31^ or not reported^33^, ^34^, ^35^, ^37^, ^42^, ^43^. In the rest, follow‐up was until discharge (median length of hospital stay less than 30 days)^24^, ^25^, ^39^, ^40^, 35 days after surgery^30^ or 7 days after surgery^29^.

In six^26^, ^28^, ^32^, ^34^, ^36^, ^41^ of the 13 studies reporting mortality relevant to the present study, mortality was reported 30 days after surgery. In two studies^25^, ^33^, mortality was reported until discharge and because median length of hospital stay was approximately 18 days^25^ and 28 days^33^, data on mortality were used in the meta‐analysis. Four studies reported mortality 30 days after discharge^20^, ^21^, ^22^, ^27^, and one study^30^ 35 days after surgery.

Seven studies^20^, ^21^, ^22^, ^24^, ^25^, ^27^, ^40^ defined malnutrition as more than 10 per cent loss of usual bodyweight in the last 6 months. Other studies determined malnutrition by means of evaluation tools such as nutritional risk screening^26^, ^32^, the nutritional risk index^39^, subjective global assessment^28^, ^33^ and the malnutrition universal screening tool^29^, or by nutritional parameters^36^. One study^23^ combined the use of evaluation tools and nutritional parameters. Six studies^30^, ^34^, ^35^, ^37^, ^38^, ^39^ did not describe how nutritional state was assessed.

A mean oral immunonutrition intake or compliance was described in 12 studies^21^, ^22^, ^25^, ^26^, ^27^, ^29^, ^30^, ^31^, ^32^, ^34^, ^36^, ^38^.

### Risk of bias

**Figure 2:**
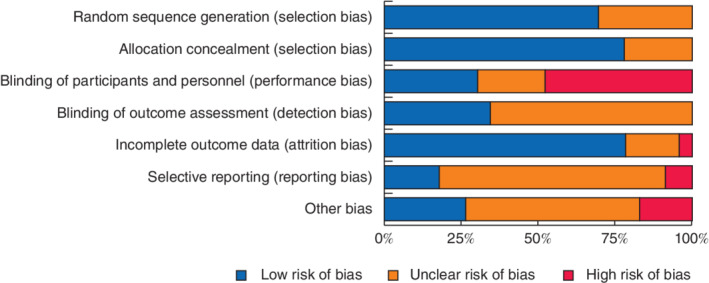
Cochrane risk‐of‐bias chart

The majority of studies used an adequate method of randomization (16 of 22, 73 per cent) and allocation (18 of 22, 82 per cent); however, the rest did not report the method of randomization and allocation and were considered to have an unclear risk of bias. High risk of performance bias was present in half of the studies (11 of 22, 50 per cent) as they were unblinded. Detection bias was unclear in a majority of studies (15 of 22, 68 per cent) and considered low in the remainder (7 of 22, 32 per cent). Risk of attrition bias was low in 18 studies (82 per cent), high in one study (5 per cent) and unclear in the rest. Risk of reporting bias was unknown in the majority of studies (17 of 22, 77 per cent), low in three studies (14 per cent) and high in two studies (9 per cent). Four trials were at high risk of other biases (*Fig*. *2*; *Fig. S1*, supporting information).

### Adverse effects

Thirteen studies^20^, ^21^, ^22^, ^24^, ^26^, ^28^, ^29^, ^34^, ^35^, ^36^, ^38^, ^39^ described adverse effects of, or tolerance to, the study intervention, of which six were limited to tolerance of tube feeding in the postoperative period. Adverse effects were mostly gastrointestinal. No statistically significant differences were seen in all but one study^28^, which found a higher incidence of postoperative diarrhoea in the immunonutrition group compared with the control group.

### Meta‐analysis

#### Primary outcome: overall infectious complications

**Figure 3:**
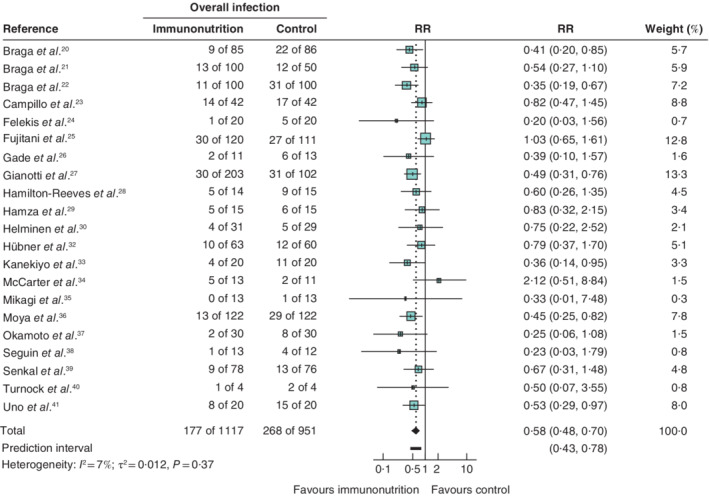
Forest plot comparing overall infectious complications in immunonutrition and control groups A random‐effects model was used for meta‐analysis. Relative risks (RRs) are shown with 95 per cent confidence intervals.

For the 21 RCTs reporting on 2068 patients, a significant effect was seen in favour of immunonutrition compared with the control group (RR 0·58, 95 per cent c.i. 0·48 to 0·70; *I* ^2^ = 7 per cent). A 95 per cent prediction interval estimated the effect in future studies to be 0·43 to 0·78 (*Fig*. *3*).

#### Secondary outcomes: surgical‐site infection and mortality

**Figure 4:**
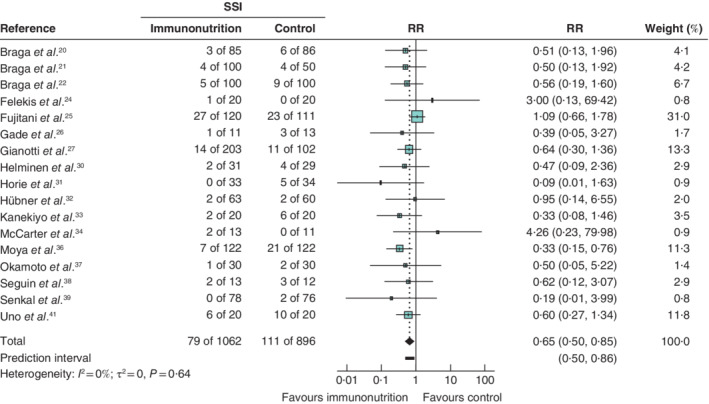
Forest plot comparing surgical‐site infection in immunonutrition and control groups A random‐effects model was used for meta‐analysis. Relative risks (RRs) are shown with 95 per cent confidence intervals. SSI, surgical‐site infection.

Of 17 studies that reported on 1958 patients, a pooled RR in favour of immunonutrition was evident (RR 0·65, 95 per cent c.i. 0·50 to 0·85; *I* ^2^ = 0 per cent). A 95 per cent prediction interval estimated the effect in future studies to be 0·50 to 0·86 (*Fig*. *4*).

**Figure 5:**
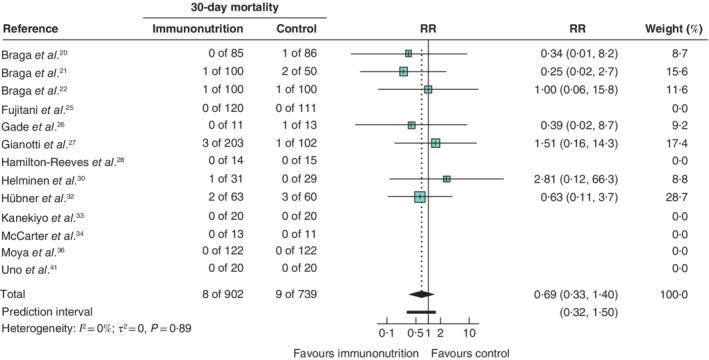
Forest plot comparing 30‐day mortality in immunonutrition and control groups A random‐effects model was used for meta‐analysis. Relative risks (RRs) are shown with 95 per cent confidence intervals.

The pooled RR for the 13 studies that reported on 30‐day mortality in 1641 patients was 0·69 (95 per cent c.i. 0·33 to 1·40; *I* ^2^ = 0 per cent), with a 95 per cent prediction interval of 0·32 to 1·50 (*Fig*. *5*).

### Exploratory analyses

Results of exploratory analyses are available as supporting information (*Figs S2–S12*).

Subgroup analysis did not show any statistically significant difference in overall infectious complications between patients receiving perioperative immunonutrition and those receiving preoperative immunonutrition (*P* = 0·070). A significant difference was found for SSI (*P* = 0·006).

No significant difference was found between studies in which the control group received active comparator *versus* no supplement (overall infectious complications: *P* = 0·282; SSI: *P* = 0·106). When the control group received active comparator, no significant difference was found between blinded and unblinded studies (overall infectious complications: *P* = 0·285; SSI: *P* = 0·925).

Subgroup analysis of different nutritional states showed no difference between the groups (overall infectious complications: *P* = 0·518; SSI: *P* = 0·847).

### Trial sequential analysis

TSA results are depicted graphically in *Figs S13–S18* (supporting information).

#### Overall infectious complications

For overall infectious complications, using a RRR of 10 per cent as the effect estimate, the naive 95 per cent c.i. was 0·48 to 0·70 (RR 0·58) and the TSA‐adjusted c.i. was 0·28 to 1·21. When using a RRR of 20 per cent as estimate, the *Z*‐curve crossed both the conventional naive boundaries and the trial sequential monitoring boundaries after six trials. The TSA‐adjusted c.i. was 0·46 to 0·73.

In the 10 per cent RRR conservative scenario TSA, the diversity‐adjusted required information size (DARIS) was not reached for benefit of immunonutrition, but TSA boundaries for benefit were crossed in the 20 per cent RRR liberal scenario.

#### Surgical‐site infection

For SSI, when using a RRR of 10 per cent as estimate, the naive 95 per cent c.i. was 0·50 to 0·85 (RR 0·65) and the TSA‐adjusted c.i. was 0·21 to 2·04. Using a RRR of 20 per cent as estimate, the *Z*‐curve crossed the conventional naive boundaries after six trials, but the trial sequential monitoring boundaries were not crossed. The TSA‐adjusted c.i. was 0·40 to 1·06.

#### 30‐day mortality

For 30‐day mortality, when using a RRR of both 10 and 20 per cent, the *Z*‐curve did not cross the conventional naive boundaries and the trial sequential monitoring boundaries could not be determined owing to lack of information. The estimate and the naive 95 per cent c.i. was 0·69 (0·33 to 1·40). The TSA‐adjusted c.i. could not be calculated.

### Publication bias

Funnel plots did not detect any significant asymmetry indicative of publication bias in studies reporting overall infectious complications or SSI (*Figs S19* and *S20*, supporting information). Egger's test gave *P* = 0·3 for overall infectious complications and *P* = 0·2 for SSI.

### GRADE assessment

All three outcomes were downgraded one level because 11 trials were unblinded, presenting a high risk of performance bias from a critical proportion of the studies.

In studies reporting on 30‐day mortality and SSI, meta‐analysis showed an imprecision that was serious enough to downgrade a level, because the optimal information size criterion was not met (*Table S5*, supporting information).

## Discussion

Meta‐analysis indicated that preoperative or perioperative immunonutrition reduced the risk of developing an infectious complication by 42 per cent and the risk of SSI by 35 per cent. The quality of evidence was moderate for overall infectious complications, mainly due to the high risk of performance bias because of unblinded studies. The quality of evidence was low for SSI, owing to the high risk of performance bias and imprecision. In the TSA assuming a RRR of 20 per cent, immunonutrition significantly reduced overall infectious complications. However, results of the other TSA analyses indicated the need for further trials. Previous meta‐analyses have restricted their scope to a particular cancer subtype, whereas the present study investigated the impact on patients undergoing surgery for solid malignant tumours of any kind.

Immunonutrition by the oral route seems preferable to tube or parenteral feeding as part of the routine optimization of patients undergoing cancer surgery. Tube feeding in the postoperative period does not comply with enhanced recovery after surgery protocols, is associated with a higher risk of pulmonary infections, and is often poorly tolerated by the patient^44^, ^45^. One study^46^ found increased infectious complications in patients who received preoperative parenteral nutrition.

In the present review, approximately half of studies did not report on adverse effects of immunonutrition. Among those that did, one found a significantly higher incidence of postoperative diarrhoea in the immunonutrition group compared with the control group.

Subgroup analysis of preoperative *versus* perioperative immunonutrition showed no significant difference for the primary outcome of overall infectious complications, although only perioperative immunonutrition was associated with a significantly reduced SSI. Further studies should examine the additional effect of continuing immunonutrition in the postoperative period.

The exploratory analysis of control groups receiving an active comparator *versus* no supplement found no statistically significant difference for the primary outcome. This could indicate that the effect of immunonutrition lies in key components that modulate the immune system, and not the basic nutritional supplementation. It could also be the result of lack of blinding in the studies that had an active comparator, although subgroup analysis did not show any significant difference between blinded and unblinded studies receiving an active comparator.

Subgroup analysis of the nutritional state of patients showed no significant difference between nutritional states, indicating that immunonutrition could improve postoperative outcomes by pharmacological rather than nutritional mechanisms. These results should be interpreted with caution though, as ten studies mixed well nourished and malnourished patients, information on nutritional state could not be obtained from four studies, and studies that reported on nutritional state did not define and measure malnutrition by the same standards.

The quality of evidence for all three outcomes was affected by the high risk of performance bias, as 11 trials were not properly blinded, including four studies in which the control group received an active comparator, where blinding would have been possible. Subgroup analysis showed no significant difference between blinded and unblinded studies with active comparators, although data were sparse.

A simulation study^47^ has shown that up to 1000 randomized patients are needed in a study to ensure balance in baseline characteristics between allocation groups. Thus, the studies included in the present review may suffer from baseline imbalances.

Meta‐analysis of all outcomes had a low *I* ^2^ value, indicating low statistical heterogeneity. However, this does not take the clinical and methodological heterogeneity into account.

There are trials of immunonutrition underway, as well as some results in patients with ovarian, bladder and thymus cancers, but the evidence is still very limited on the effect of immunonutrition in a variety of cancer subtypes^28^, ^43^, ^48^. Different cancer subtypes involve operations of highly variable extent, with their own risks of complications and mortality.

There was inadequate compliance among the studies included in the present review. Only 12 of the 24 studies reported mean oral immunonutrition intake or compliance, and compliance could be a challenge to the possibility of assessing a dose–response relationship.

In the studies reporting on mortality, the number of events was small. Thirty‐day mortality after elective surgery for the cancer types in the included studies ranges from 3·8 to 8·8 per cent^49^, but in the meta‐analysis it was only 0·9 per cent in the immunonutrition group and 1·2 per cent in the control group. Of the included studies, only 11 reported 30‐day mortality. There is need for studies describing 30‐day outcomes and the long‐term impact of immunonutrition.

PubMed, Cochrane Library and Embase were the only databases searched, and some literature may have been overlooked. It was beyond the scope of this review to make a cost–benefit analysis, although other analyses^39^, ^50^, ^51^, ^52^ have shown better cost‐effectiveness in patients receiving perioperative or preoperative immunonutrition compared with standard care.

Guidelines^53^ suggest that preoperative oral immunonutrition should be given 5–7 days before surgery at a dose of 500–1000 ml per day. The doses and duration of immunonutrition in the studies included in this review differed, and this might influence the study findings. Only one study^54^ investigated the effects of different doses of immunonutrition, and found no difference between receiving 500 or 1000 ml/day. An important aspect of gaining knowledge on the optimal dose of oral immunonutrition is biochemical demonstration of effective uptake of the key components of immunonutrition, but this has not been reported widely.

Future trials should produce high‐quality evidence, and these trials should be properly blinded. There is an urgent need for proper reporting of side‐effects. Trials comparing preoperative with perioperative immunonutrition should be prioritized, and perioperative immunonutrition in these trials should be given orally, both before and after surgery. Dose–response studies are needed to clarify the optimal dose of immunonutrition. All of these issues indicate a need for multicentre pragmatic trials with long‐term follow‐up.

## Disclosure

The authors declare no conflict of interest.

## Supporting information


**Appendix** **S1**: Supporting information
