# Supplementary material for: Impact of oral preoperative and perioperative immunonutrition on postoperative infection and mortality in patients undergoing cancer surgery: systematic review and meta‐analysis with trial sequential analysis
Source: BJS Open. 2020 Jun 23;4(5):764–75. doi: 10.1002/bjs5.50314 (PMC7528521; doi:10.1002/bjs5.50314)
Supplement: Supplementary file 1 — Appendix S1: Supporting information [file BJS5-4-764-s001.docx]

**BJS5_50314**

Impact of oral preoperative and perioperative immunonutrition on postoperative infection and mortality in patients undergoing cancer surgery: systematic review and meta-analysis with trial sequential analysis

**F. Buzquurz, R. D. Bojesen, C. Grube, M. T. Madsen and I. Gögenur**

## Appendix S1 Search strategy

## PubMed

((((cancer*) OR (neoplasm*) OR (“tumor*”) OR (“carcinoma*”) OR (“oncologic surgery”))) OR ("Neoplasms"[Mesh])) AND (((“immunonutrition*”) OR (“Nutritional Support”) OR (”Arginine”) OR (”Glutamine”) OR (“Omega-3 fatty acid”) OR (“n-3 Fatty Acids”) OR (“Nucleotide”) OR (“RNA”) OR (“Dietary supplements”)) OR ((("Nutritional Support"[Mesh]) OR ("Arginine"[Mesh]) OR ("Glutamine"[Mesh]) OR ("Nucleotides"[Mesh]) OR ("RNA"[Mesh]) OR ("Fatty Acids, Omega-3"[Mesh]) OR ("Dietary supplements”[Mesh]))))) AND (((surgery) OR (preoperative) OR (perioperative)) OR ((("Surgical Procedures, Operative"[Mesh]) OR ("General Surgery"[Mesh]) OR ("surgery" [Subheading]))))) AND (((Survival) OR (Mortality) OR (Infection*) OR (Complication*)) OR (("Mortality"[Mesh]) OR ("Postoperative complications”[Mesh]) OR ("Infection"[Mesh])))) AND ((((Oral*) OR (Peroral) OR (Enteral) OR (drink*)) OR ("Enteral Nutrition"[Mesh]) OR ("Administration, Oral"[Mesh])))

**Appendix S2** Detailed description of trial sequential analysis and DARIS table

Trial sequential analysis (TSA) was applied to the current review to address the risk of random error associated with sparse data and/or multiple testing^1-5^. Analog to sample size calculations for randomised clinical trials, the TSA estimates the “required information size” (RIS) which is the total number of patients needing to be included into a meta-analysis in order to confirm or reject an effect of a given intervention. TSA can be seen as the equivalent of an interim-analysis in a randomised clinical trial where multiple test are performed as is the case with a cumulative meta-analysis^5^.

To prevent post hoc data-driven testing, several criteria must be defined a priori to the TSA is run. The criteria consist of: proportion of events in the control group for a binary outcome (or standard deviation for a continues outcome) (Pc); a realistic relative risk reduction (RRR) for a binary outcome (or minimal clinical important difference (MCID) for a continues outcome) between groups; alpha; beta; and diversity (D^2^ – estimated by the TSA model)^3,4^.

In the current review, these prespecified criteria are presented in a DARIS table. Outcomes in the current review were dichotomous and the proportion of events in control groups were based on the frequency showed within the current review. A RRR of 10% was chosen pragmatically to represent a conservative intervention effect. In a sensitivity analysis, a RRR of 20% was chosen to represent an liberal intervention effect. An alpha level of 5% was chosen for the primary outcome and 3.6% for secondary outcomes (two comparisons) as per the guidelines described by Jakobsen and colleagues^6^. A beta of 10% (i.e. power 90%) was chosen^7^ and D^2^ a measure of between study heterogeneity was calculated by the TSA model (<http://www.ctu.dk/tsa/>). Moderate heterogeneity was expected, hence, the TSA analyses were all performed using random effect models applying DerSimonian-Laird (DL) tau estimator as in the primary meta-analysis.

## DARIS table

| **Analysis** | **Pc** | **RRR^a^** | **Alpha** | **Beta** | **D^2,b^** |
| --- | --- | --- | --- | --- | --- |
| **Overall infectious complications** | 28.18% | 10% | 5% | 10% | Model estimate |
| **Surgical site infection** | 12.39% | 10% | 3,6% | 10% | Model estimate |
| **30 day mortality** | 1.219% | 10% | 3,6% | 10% | Model estimate |

DARIS indicates diversity-adjusted required information size; RRR, relative risk reduction. DL, DeSimonian-Laird. a. 20% RRR will be presented as a liberal treatment effect. b. Analysis run using random model (DL $\tau^{2}$ estimator).

## Table S1 Baseline characteristics of prospective cohort studies

| **Reference** | **Country** | **Sample size** | **Anatomical site** | **Male/female distribution** | **Nutritional state** | **Duration of follow-up** |
| --- | --- | --- | --- | --- | --- | --- |
| Rowan et al. 2016^8^ | USA | 195 | Head/neck | 142/53 | NS | NS |
| Xin et al. 2016^9^ | China | 244 | Thymus | 115/129 | Well-nourished | NS |

NS indicates Not stated

## Table S2 Intervention characteristics and result of prospective cohort studies

| **Reference** | **Preop./postop duration (days)** | **Preop. IN dose** | **Postop. IN dose** | **Nutrients in immunonutrition** | **Control group** | **Overall infectious complications** | **Surgical site infection** |
| --- | --- | --- | --- | --- | --- | --- | --- |
| Rowan et al. 2016^8^ | 5/5 | 700 mL/day | NS | Arginine, omega-3, RNA | No supplement | NS | No statistical significance^a^ |
| Xin et al. 2016^9^ | 7/7 | NS | NS | Arginine, omega-3, RNA | No supplement | Statistical significance favoring IN^b^ | No statistical significance^a^ |

Preop indicates preoperative; postop., postoperative, IN, Immunonutrition; NS, not stated. a. p > 0.05. b. p < 0.05.

## Table S3 Overview of contacted authors

| **Contacted author** | **Article** | **Requested information** | **Response?** |
| --- | --- | --- | --- |
| Gade et al. 2016^10^ | The effect of preoperative oral immunonutrition on complications and length of hospital stay after elective surgery for pancreatic cancer-A randomized controlled trial | Outcomes on malignant cases only | Yes |
| Helminen et al. 2007^11^ | Immunonutrition in elective gastrointestinal surgery patients | Outcomes on malignant cases only and nutritional state of patients | Yes |
| Hübner et al.2012^12^ | Preoperative immunonutrition in patients at nutritional risk: results of a double-blinded randomized clinical trial | Outcomes on malignant cases only | Yes |
| Hamilton-Reeves et al. 2015^13^ | Effects of Immunonutrition for Cystectomy on Immune Response and Infection Rates: a Pilot Randomized Controlled Clinical Trial | Nutritional state of patients | Yes |
| Mikagi et al. 2010^14^ | Effect of preoperative immunonutrition in patients undergoing hepatectomy; a randomized controlled trial | Period of follow-up and nutritional state of patients | No |
| Okamoto et al. 2009^15^ | Attenuation of the systemic inflammatory response and infectious complications after gastrectomy with preoperative oral arginine and omega-3 fatty acids supplemented immunonutrition | Period of follow-up and nutritional state of patients | No |
| Seguin et al. 2016^16^ | Effect of a Perioperative Nutritional Supplementation with Oral Impact in Patients undergoing Hepatic Surgery for Liver Cancer: a Prospective, Placebo-Controlled, Randomized, Double-Blind Study | Period of follow-up | No |
| Uno et al. 2016^17^ | Immunonutrition suppresses acute inflammatory responses through modulation of resolvin E1 in patients undergoing major hepatobiliary resection | Nutritional state of patients | Yes |

## Table S4 Baseline characteristics

| **Reference** | **Male/female distribution** | **Nutritional state** | **Duration of follow-up** |
| --- | --- | --- | --- |
| Braga et al. 1999^18^ | 106/65 | Mixed | 30 days after discharge |
| Braga et al. 2002 (1)^19^ | 84/66 | Malnourished | 30 days after discharge |
| Braga et al. 2002 (2) ^20^ | 118/82 | Mixed | 30 days after discharge |
| Campillio et al. 2017^21^ | 58/26 | Mixed | 30 days after discharge |
| Felekis et al. 2010^22^ | 36/4 | Mixed | Until discharge (Median LOS 12 days) |
| Fujitani et al. 2012^23^ | 181/63 | Well-nourished | Until discharge  (Median LOS 17/18 days) |
| Gade et al. 2016^10^ | 11/13 | Mixed | 30 days after surgery |
| Gianotti et al. 2002^24^ | 166/139 | Well-nourished | 30 days after discharge |
| Hamilton-Reeves et al. 2015^13^ | 29/0 | NS | 30 days after surgery |
| Hamza et al. 2015^25^ | 20/17 | Malnourished | 7 days after surgery |
| Helminen et al. 2007^11^ | 42/18 | NS | 35 after surgery |
| Horie et al. 2006^26^ | 43/24 | Well-nourished | 30 days after discharge |
| Hübner et al 2012^12^ | 73/50 | Malnourished | 30 days after surgery |
| Kanekiyo et al. 2018^27^ | 32/8 | Mixed | NS  (In-hospital mortality, median LOS 28 days) |
| McCarter et al. 1998^28^ | 21/17 | NS | NS  (Mortality – 30 days after surgery) |
| Mikagi et al. 2010^14^ | 18/8 | NS | NS |
| Moya et al. 2016^29^ | 131/113 | Well-nourished | 30 days after surgery |
| Okamoto et al. 2009^15^ | 42/18 | NS | NS |
| Seguin et al. 2016^16^ | 31/4 | NS | 30 days after surgery |
| Senkal et al. 1999^30^ | 100/54 | Mixed | Until discharge  (Mean LOS 22,2/25,8 days) |
| Turnock et al. 2013^31^ | 6/2 | Well-nourished | Until discharge  (Median LOS 10/21,5 days) |
| Uno et al. 2016^17^ | 25/15 | NS | 30 days after surgery |

LOS, Length of hospital stay; NS, Not stated; POD, Postoperative day.

## Fig. S1 Risk-of-bias summary


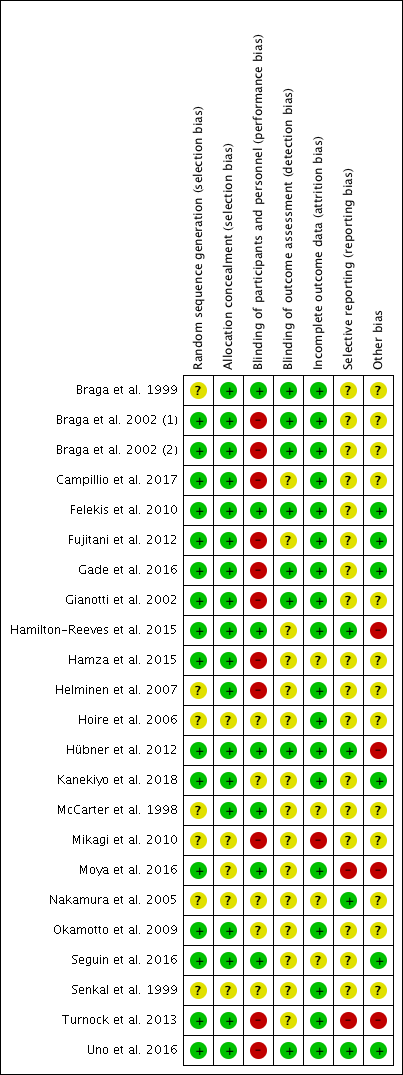


## Fig. S2 Subgroup analysis of preoperative *versus* perioperative immunonutrition: overall infectious complications


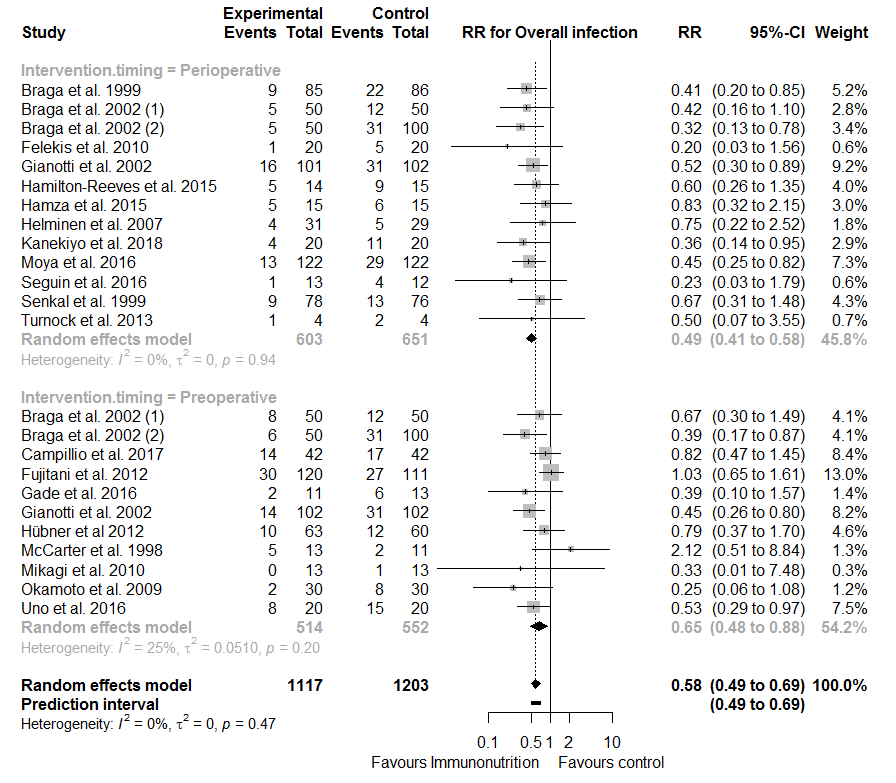


Subgroup analysis did not show any statistically significant difference between patients receiving perioperative immunonutrition vs. preoperative immunonutrition (P = 0.07).

## Fig. S3 Subgroup analysis of preoperative *versus* perioperative immunonutrition: surgical-site infection


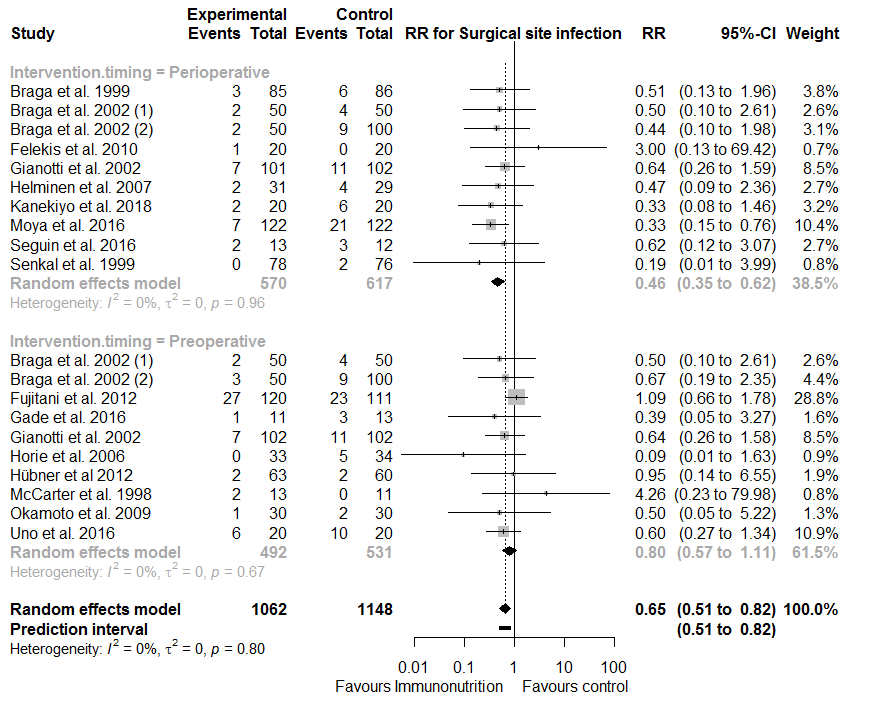


A statistically significant difference was found between groups of patients receiving perioperative immunonutrition and preoperative immunonutrition (P = 0.0057). Perioperative administration was associated with better outcome (RR 0.46, 95% Cl 0.35-0.62).

## Fig. S4 Subgroup analysis by control group: overall infectious complications


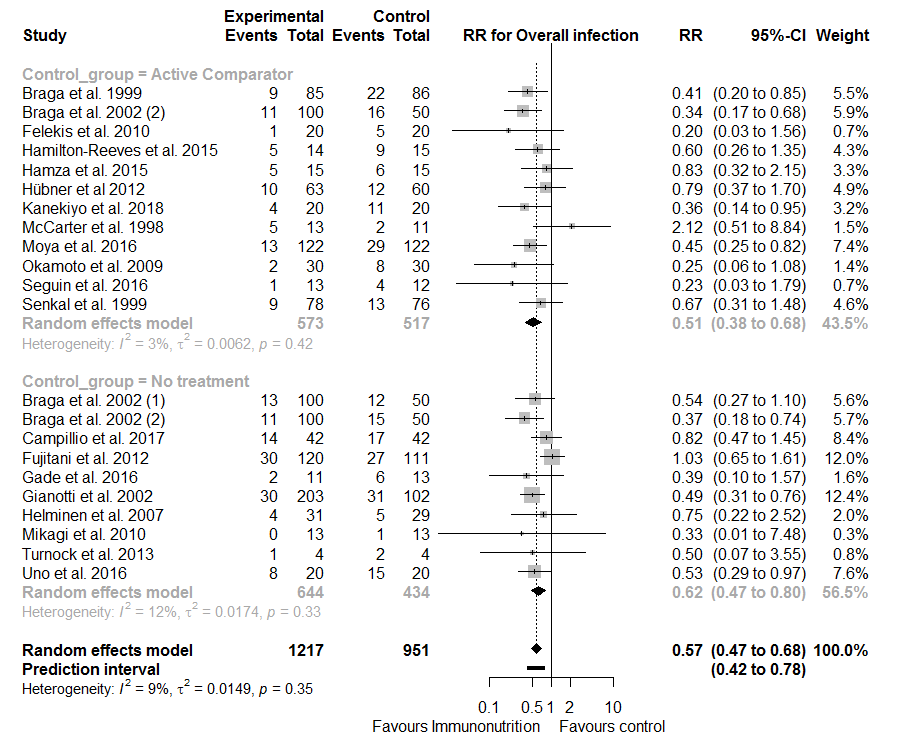


There was no statistically significant difference between studies with the two different control groups (P = 0.28).

## Fig. S5 Subgroup analysis by control group: surgical-site infection


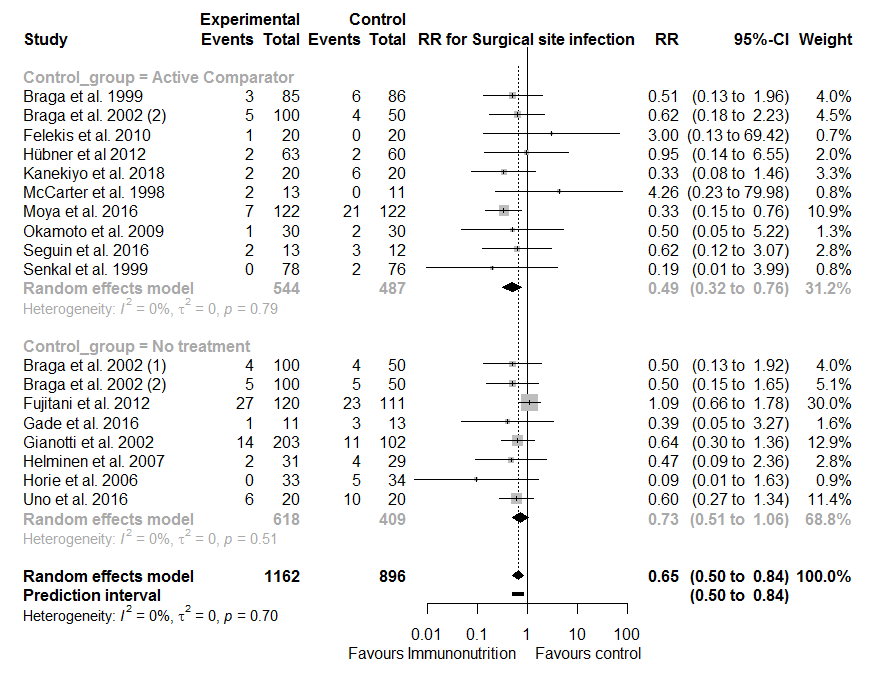


There was a statistically significant difference between the two different control groups (P = 0.11). Studies in which the control group received placebo was associated with better outcome.

## Fig. S6 Subgroup analysis of blinding in active comparator controls: overall infectious complications


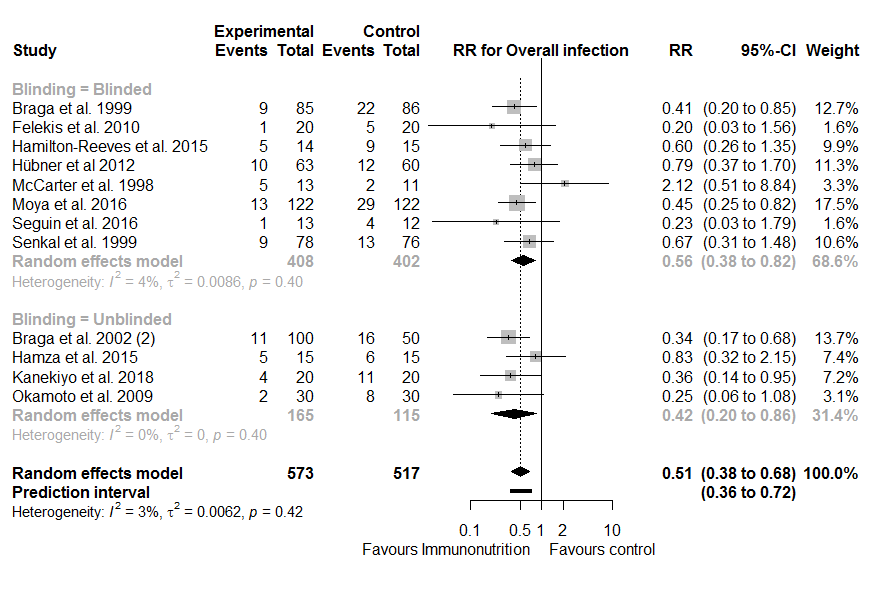


There was no statistically significant difference in overall infectious complications between blinded and unblinded studies in with active comparator (P = 0.29).

## Fig. S7 Subgroup analysis of blinding in active comparator controls: surgical-site infection


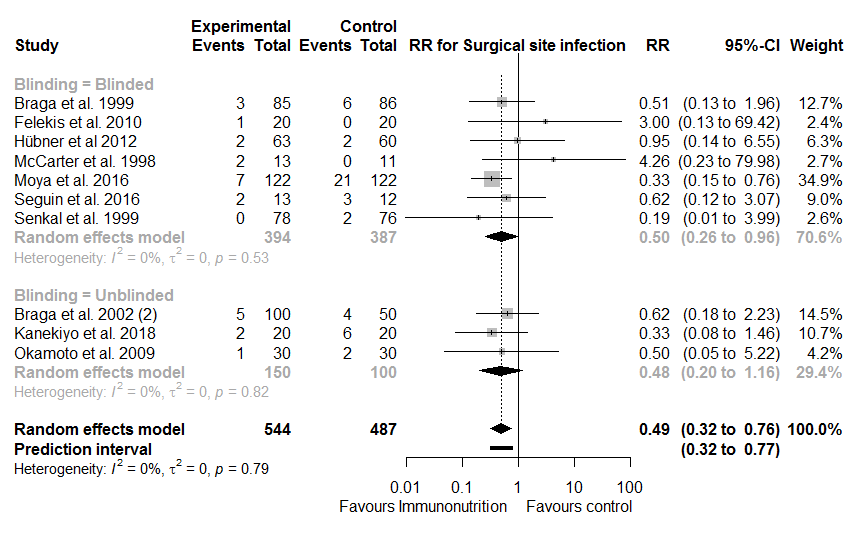


There was no statistically significant difference in surgical site infections between blinded and unblinded studies in with active comparator (P = 0.93).

## Fig. S8 Subgroup analysis by nutritional state: overall infectious complications


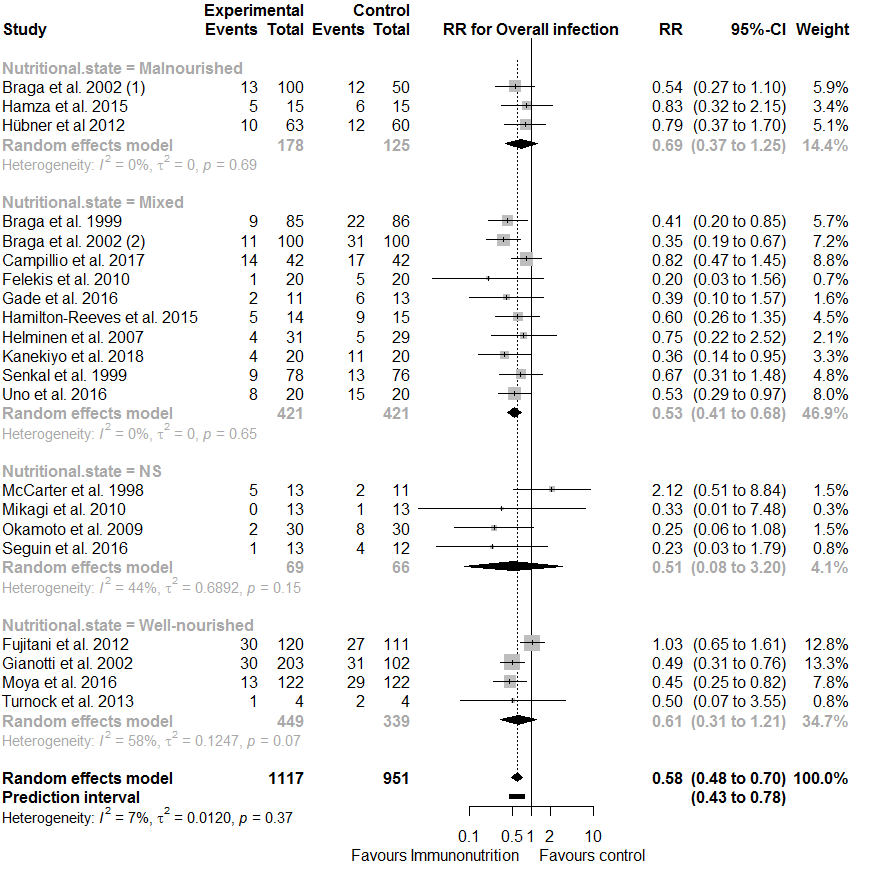


Subgroup analysis of different nutritional states showed no statistically significant difference (P = 0.518)

## Fig. S9 Subgroup analysis by nutritional state: surgical-site infection


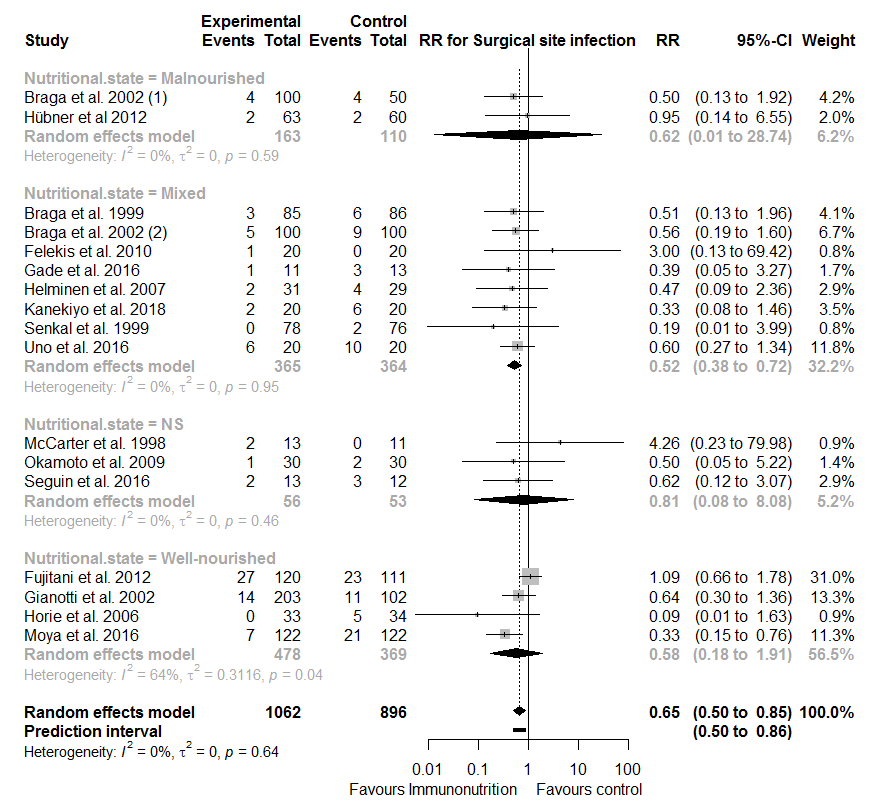

Subgroup analysis of different nutritional states showed no statistically significant difference (P = 0.847).

## Fig. S10 Fixed model sensitivity analysis: overall infectious complications


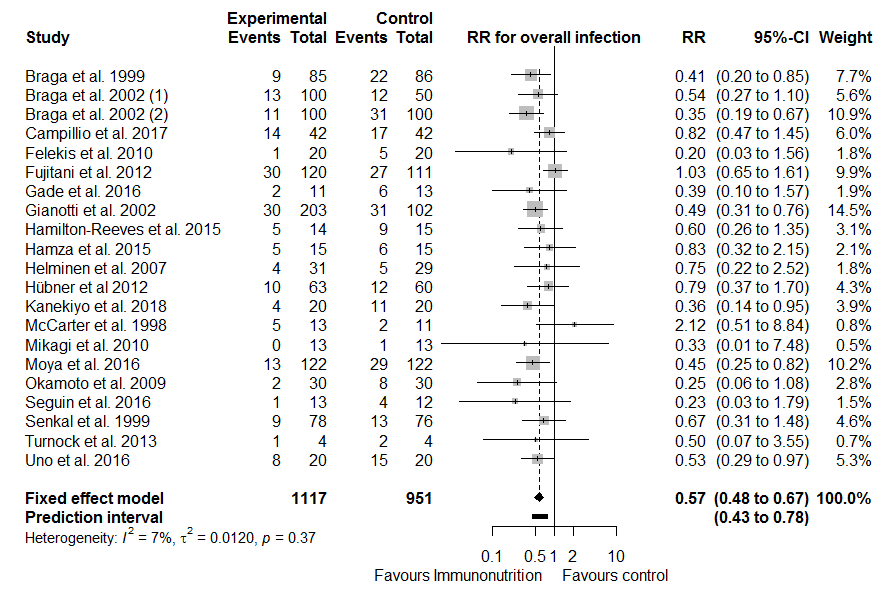


Applying the fixed effect model is largely congruent with the random effects models since analysis shows presence of low statistical heterogeneity.

## Fig. S11 Fixed model sensitivity analysis: surgical-site infection


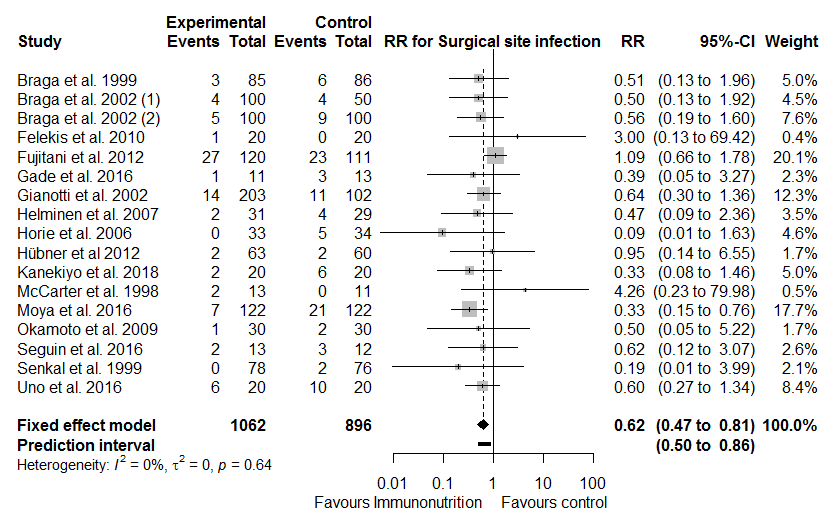


Applying the fixed effect model is largely congruent with the random effects models since analysis shows presence of low statistical heterogeneity.

## Fig. S12 Fixed model sensitivity analysis: 30-day mortality


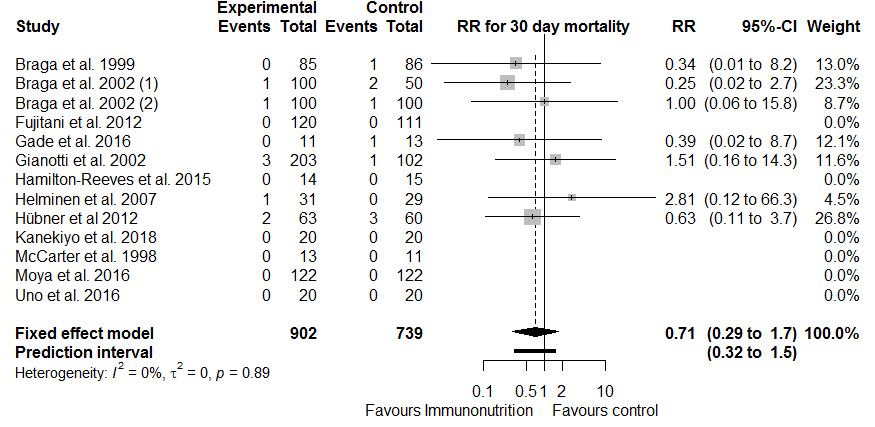


Applying the fixed effect model is largely congruent with the random effects models since analysis shows presence of low statistical heterogeneity.

##
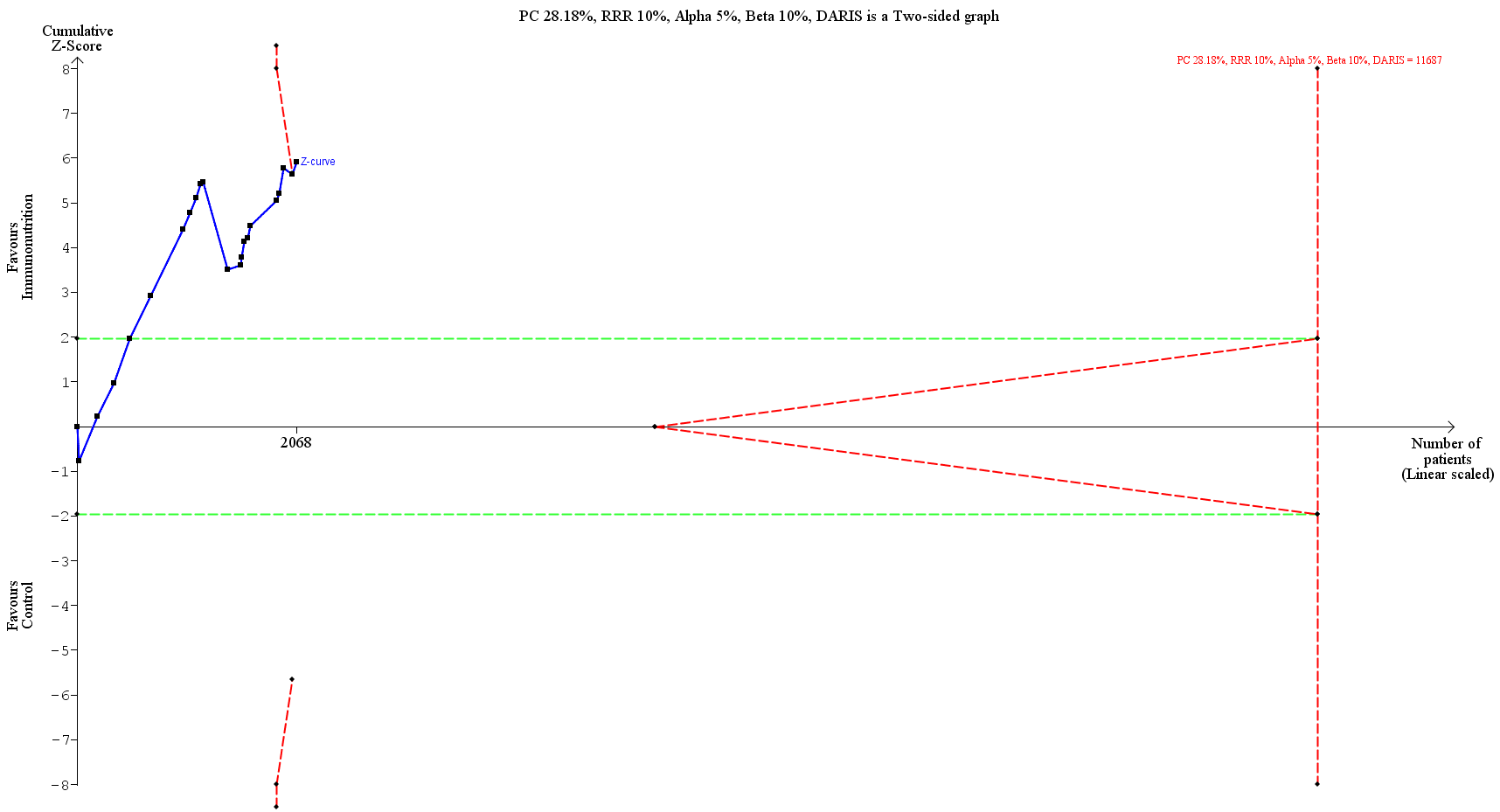
Fig. S13 Trial sequential analysis with estimated 10 per cent relative risk reduction: overall infectious complications

TSA of the effect of immunonutrition vs. control on overall infectious complications in patients undergoing cancer surgery. An estimated DARIS of 11.687 patients to detect or discard a relative risk reduction of 10% was calculated by random-effect TSA, an alpha of 0.05, and a beta of 0.10. The results show that after 4 trials, the z-curve crossed the conventional naïve 5% (CON) boundaries but it did not cross the trial sequential monitoring boundaries after 21 studies. TSA indicates Trial Sequential Analysis; DARIS, diversity-adjusted required information size; RRR, relative risk reduction. TSA, Trial Sequential Analysis; DARIS, diversity-adjusted required information size; RRR, relative risk reduction.

##
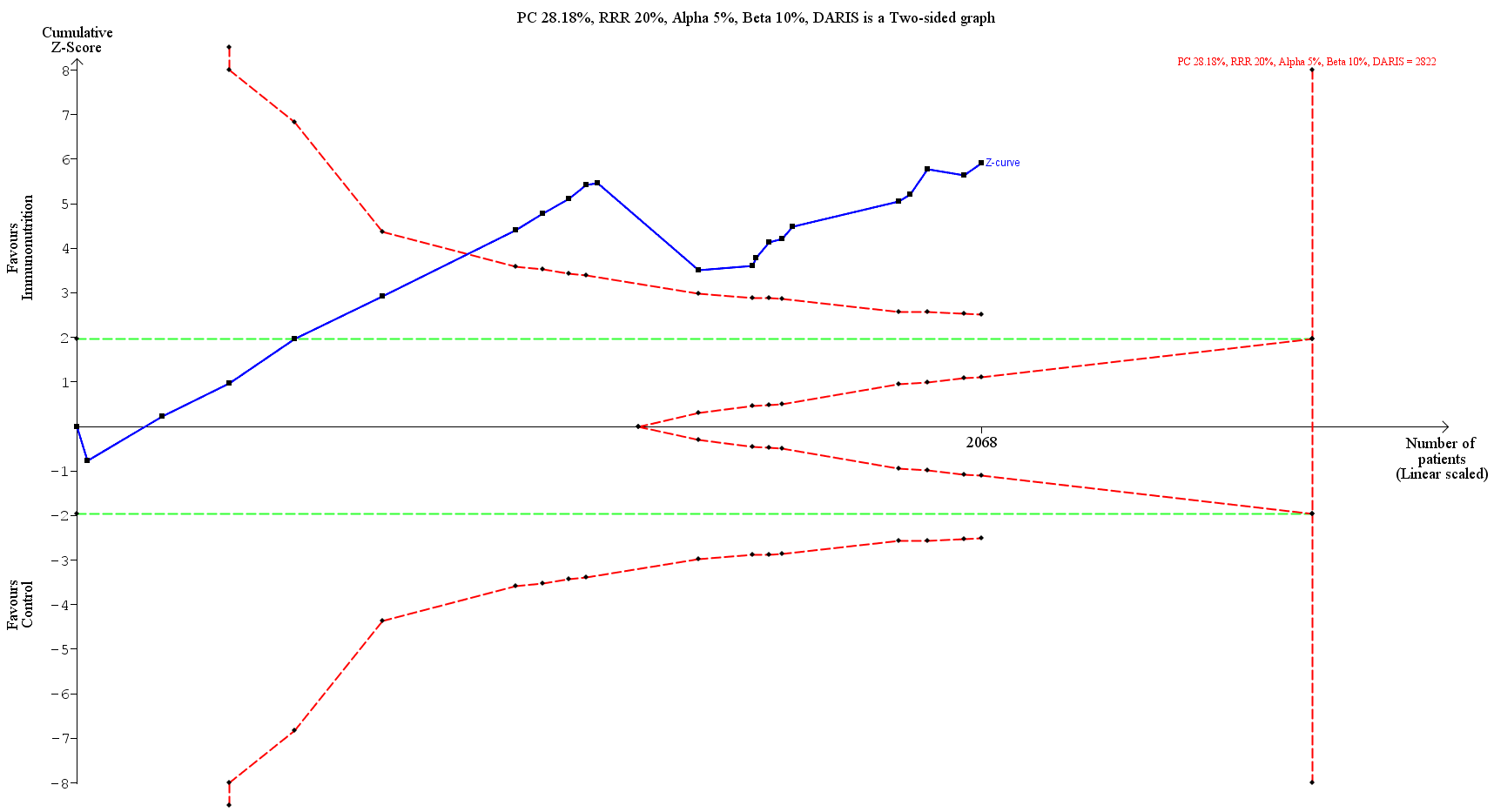
Fig. S14 Trial sequential analysis with estimated 20 per cent relative risk reduction: overall infectious complications

TSA of the effect of immunonutrition vs. control on overall infectious complications in patients undergoing cancer surgery. An estimated DARIS of 2.822 patients to detect or discard a RRR of 20% was calculated by random-effect TSA, an alpha of 0.05, and a beta of 0.10. The z-curve crossed both the CON boundaries and the trial sequential monitoring boundaries after six trials. TSA indicates Trial Sequential Analysis; DARIS, diversity-adjusted required information size; RRR, relative risk reduction.

## Fig. S15 Trial sequential analysis with estimated 10 per cent relative risk reduction: surgical-site infection


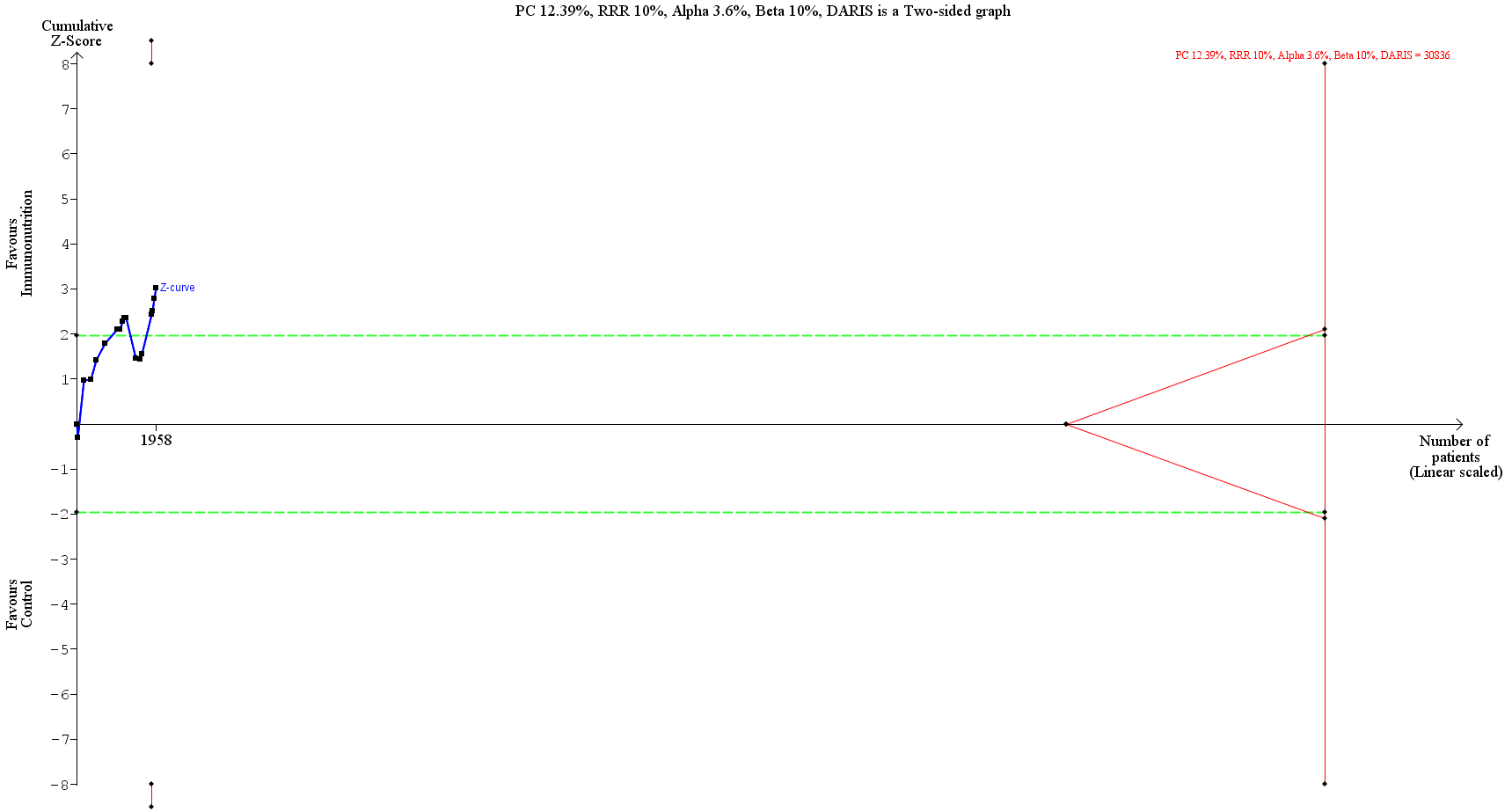


TSA of the effect of immunonutrition vs. control on surgical site infection in patients undergoing cancer surgery. An estimated DARIS of 30.836 patients to detect or discard a RRR of 10% was calculated by random-effect TSA, an alpha of 0.036, and a beta of 0.10.The z-curve crosses the CON boundaries after 6 trials, however, the trial sequential monitoring boundaries were not crossed after 17 studies. TSA indicates Trial Sequential Analysis; DARIS, diversity-adjusted required information size; RRR, relative risk reduction.

## Fig. S16 Trial sequential analysis with estimated 20 per cent relative risk reduction: surgical-site infection


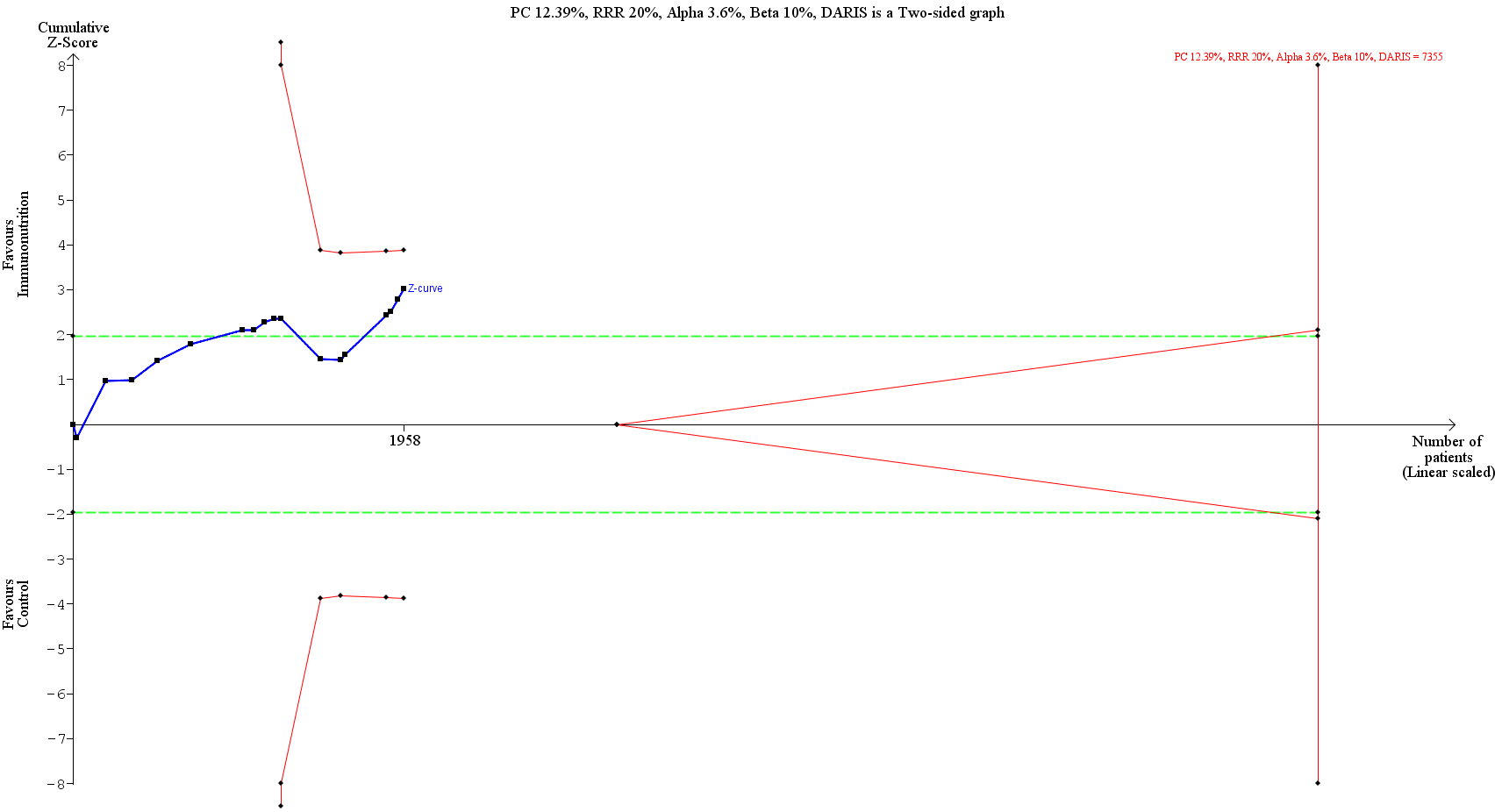


TSA of the effect of immunonutrition vs. control on surgical site infection in patients undergoing cancer surgery. An estimated DARIS of 7,355 patients to detect or discard a RRR of 20% was calculated by random-effect TSA, an alpha of 0.036, and a beta of 0.10. The z-curve crossed the CON boundaries after 6 trials, however the trial sequential monitoring boundaries were not crossed after 17 trials. TSA indicates Trial Sequential Analysis; DARIS, diversity-adjusted required information size; RRR, relative risk reduction.

## Fig. S17 Trial sequential analysis with estimated 10 per cent relative risk reduction: 30-day mortality


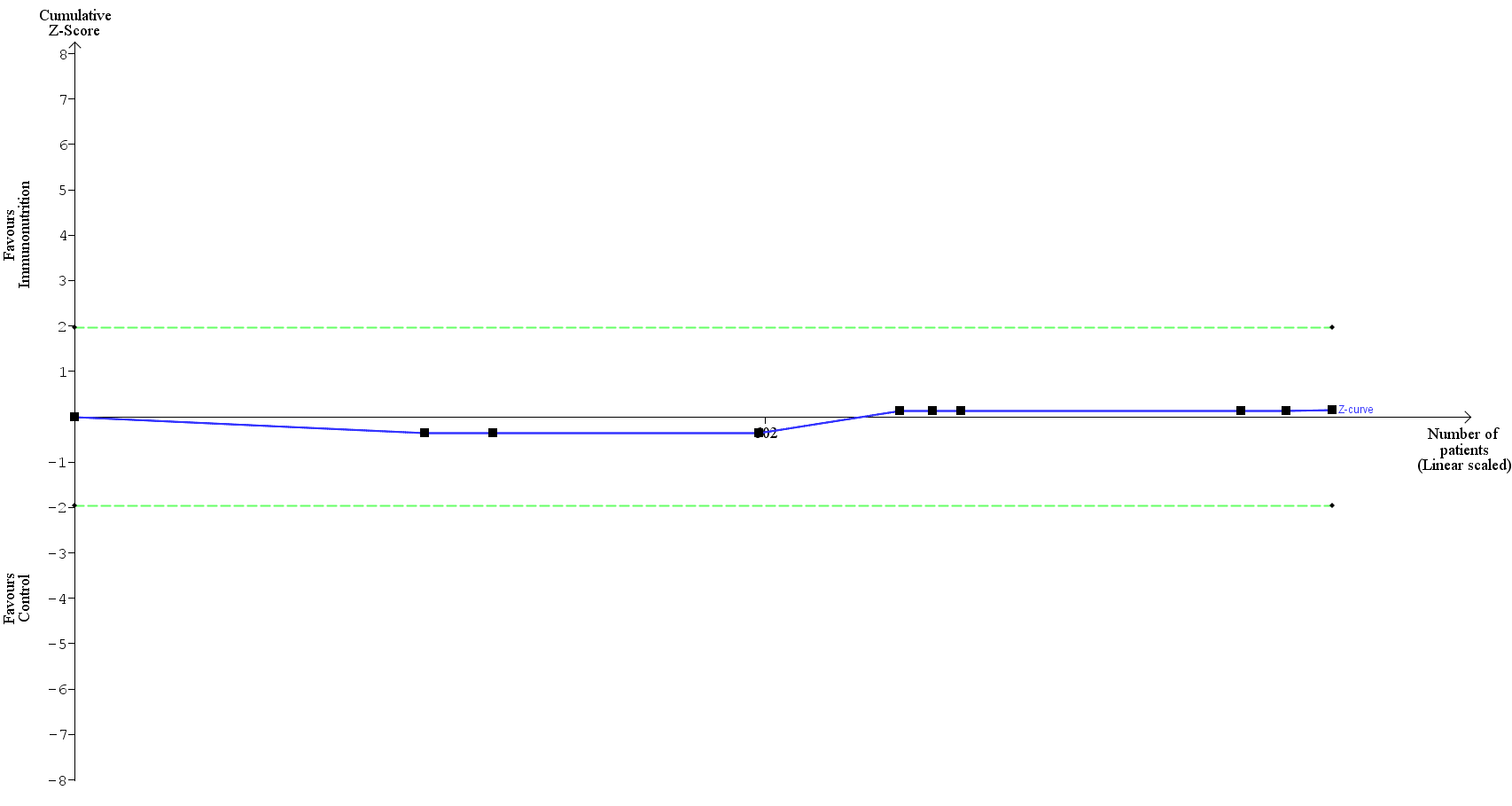


TSA of the effect of immunonutrition vs. control on 30-day mortality in patients undergoing cancer surgery. An estimated DARIS of 363.522 patients to detect or discard a RRR of 10% was calculated by random-effect TSA, an alpha of 0.36, and a beta of 0.10. Zero-event handled in TSA with empirical mode and added 0.01 to each group in events. After 13 trials the z-curve did not cross the CON boundaries and the trial sequential monitoring boundaries could not be computed due to lack of information. TSA indicates Trial Sequential Analysis; DARIS, diversity-adjusted required information size; RRR, relative risk reduction.

## Fig. S18 Trial sequential analysis with estimated 20 per cent relative risk reduction: 30-day mortality


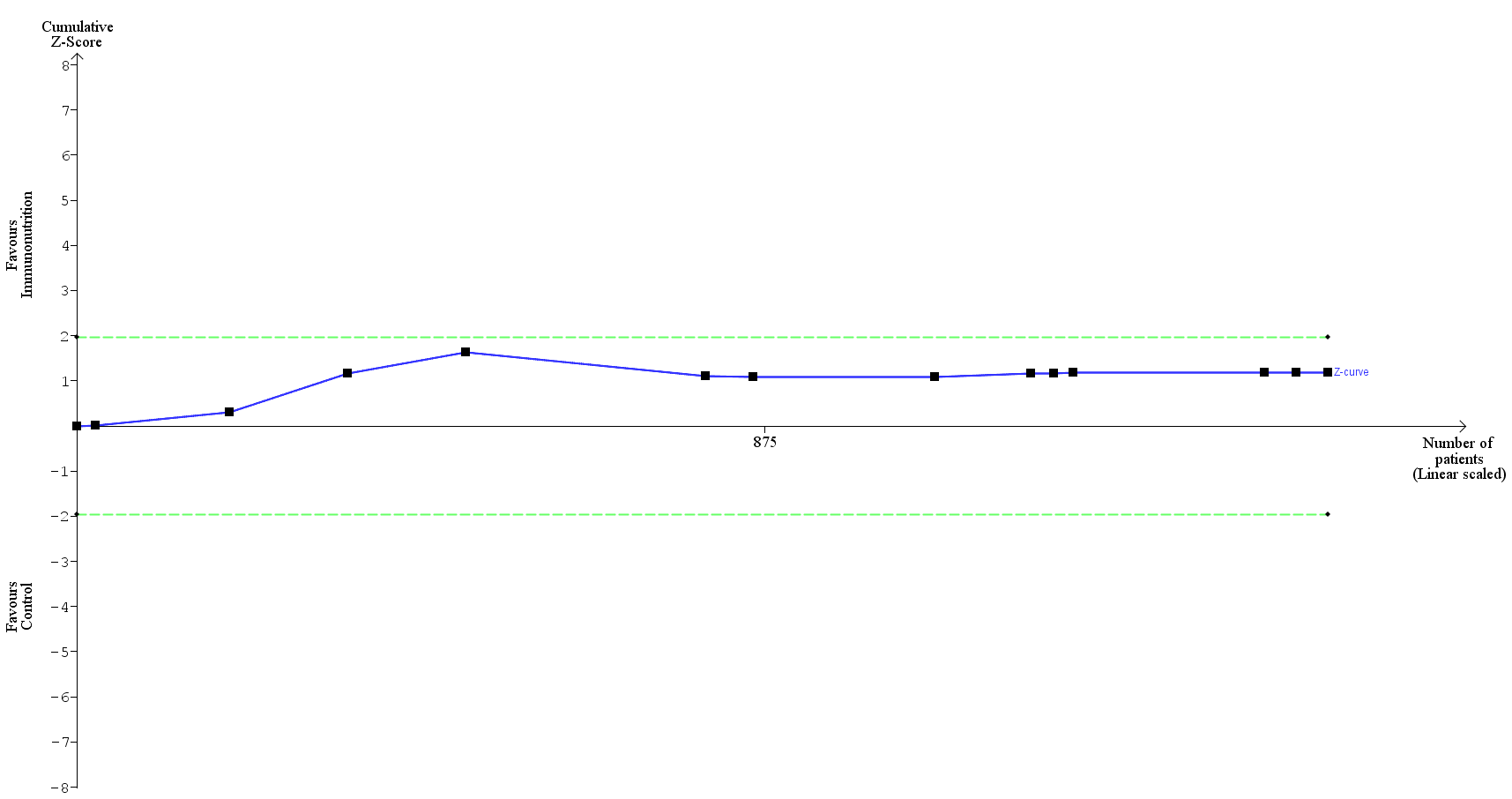


TSA of the effect of immunonutrition vs. control on 30-day mortality in patients undergoing cancer surgery. An estimated DARIS of 86,223 patients to detect or discard a RRR of 20% was calculated by random-effect TSA, an alpha of 0.36, and a beta of 0.10. Zero-event handled in TSA with empirical mode and added 0.01 to each group in events. After 13 trials the z-curve did not cross the CON boundaries and the trial sequential monitoring boundaries could not be computed due to lack of information. TSA indicates Trial Sequential Analysis; DARIS, diversity-adjusted required information size; RRR, relative risk reduction.

## Fig. S19 Funnel plot of studies reporting overall infectious complications


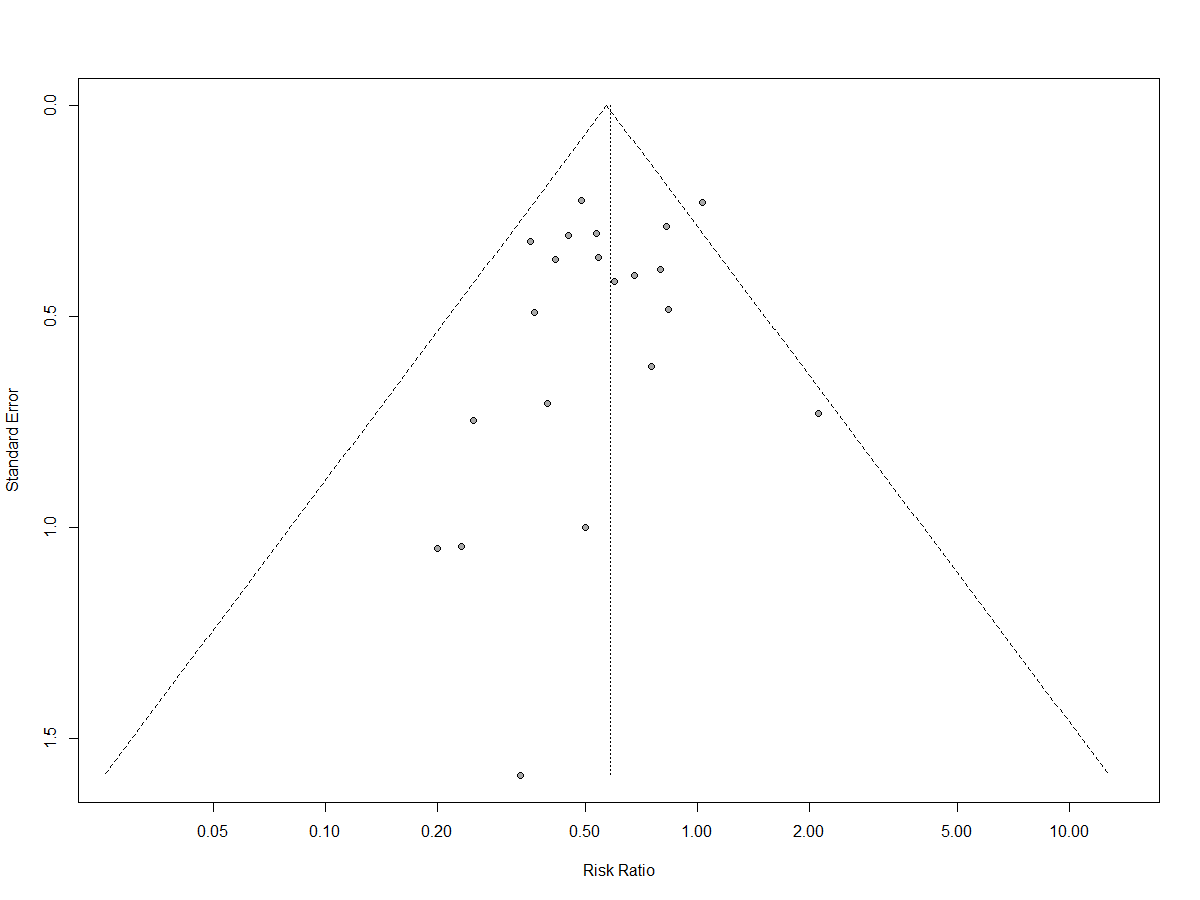


Funnel plots did not detect any significant asymmetry indicative of publication bias in studies reporting on overall infectious complications.

## Fig. S20 Funnel plot of studies reporting surgical-site infection


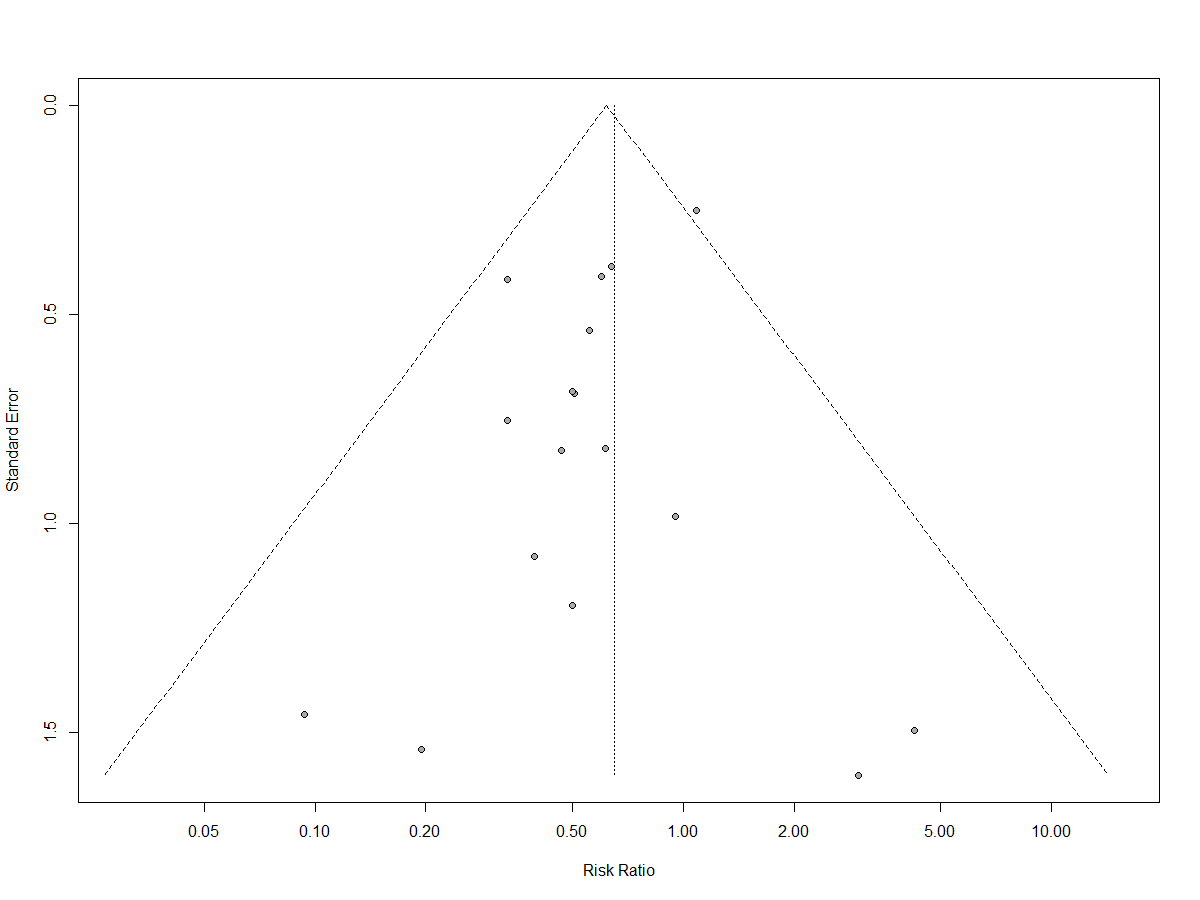


Funnel plots did not detect any significant asymmetry indicative of publication bias in studies reporting on surgical site infections.

## Table S5 GRADE assessment

| **Certainty assessment** | | | | | | | **No of patients** | | **Effect** | | **Certainty** |
| --- | --- | --- | --- | --- | --- | --- | --- | --- | --- | --- | --- |
| **No of studies** | **Study design** | **Risk of bias** | **Inconsistency** | **Indirectness** | **Imprecision** | **Other considerations** | **Immunonutrition** | **no immunonutrition** | **Relative (95% CI)** | **Absolute (95% CI)** |  |
| 21 | randomised trials | serious ^a^ | not serious | not serious | not serious | none | 177/1117 (15.8%) | 268/951 (28.2%) | **RR 0.58** (0.48 to 0.70) | **118 fewer per 1.000** (from 147 fewer to 85 fewer) | ⨁⨁⨁◯ MODERATE |
| 17 | randomised trials | serious ^a^ | not serious | not serious | Serious^b^ | none | 79/1062 (7.7%) | 111/896 (12.4%) | **RR 0.65** (0.50 to 0.85) | **43 fewer per 1.000** (from 62 fewer to 19 fewer) | ⨁⨁◯◯ LOW |
| 13 | randomised trials | serious ^a^ | not serious | not serious | Serious^b^ | none | 8/902 (0.9%) | 9/739 (1.2%) | **RR 0.69** (0.33 to 1.4) | **4 fewer per 1.000** (from 8 fewer to 5 more) | ⨁⨁◯◯ LOW |

CI indicates Confidence interval; RR: Risk ratio. a, Majority of studies were unblinded. b. Optimal information size criterion was not met.

**References**

1. Higgins J, Green S, eds. *Cochrane Handbook for Systematic Reviews of Interventions*. Version 5.1.0. The Cochrane Collaboration. <http://handbook-5-1.cochrane.org/>. Updated March 2011. Accessed June 15, 2019.

2. Thorlund K, Devereaux PJ, Wetterslev J, et al. Can trial sequential monitoring boundaries reduce spurious inferences from meta-analyses? *Int J Epidemiol.* 2009;38(1):276-286. doi: 10.1093/ije/dyn179

3. Wetterslev J, Thorlund K, Brok J, Gluud C. Trial sequential analysis may establish when firm evidence is reached in cumulative meta-analysis. *J Clin Epidemiol.* 2008;61(1):64-75. doi: 10.1016/j.jclinepi.2007.03.013

4. Wetterslev J, Thorlund K, Brok J, Gluud C. Estimating required information size by quantifying diversity in random-effects model meta-analyses. *BMC Med Res Methodol.* 2009;9:86. doi: 10.1186/1471-2288-9-86

5. Wetterslev J, Jakobsen JC, Gluud C. Trial Sequential Analysis in systematic reviews with meta-analysis. *BMC Med Res Methodol.* 2017;17(1):39. doi: 10.1186/s12874-017-0315-7

6. Jakobsen JC, Wetterslev J, Winkel P, Lange T, Gluud C. Thresholds for statistical and clinical significance in systematic reviews with meta-analytic methods. *BMC Med Res Methodol.* 2014;14(1):120. doi: 10.1186/1471-2288-14-120

7. Castellini G, Nielsen EE, Gluud C. Comment on: "Cell therapy for heart disease: Trial sequential analyses of two cochrane reviews". *Clin Pharmacol Ther.* 2017;102(1):21-24. doi: 10.1002/cpt.536

8. Rowan NR, Johnson JT, Fratangelo CE, Smith BK, Kemerer PA, Ferris RL. Utility of a perioperative nutritional intervention on postoperative outcomes in high-risk head & neck cancer patients. *Oral Oncol.* 2016;54:42-46. doi: 10.1016/j.oraloncology.2016.01.006

9. Xin Y, Cai H, Wu L, Cui Y. The Effect of Immunonutrition on the Postoperative Complications in Thymoma with Myasthenia Gravis. *Mediators Inflamm.* 2016;2016. doi: 10.1155/2016/8781740

10. Gade J, Levring T, Hillingso J, Hansen CP, Andersen JR. The effect of preoperative oral immunonutrition on complications and length of hospital stay after elective surgery for pancreatic cancer-A randomized controlled trial. *Nutr Cancer.* 2016;68(2):225-233. doi: 10.1080/01635581.2016.1142586

11. Helminen H, Raitanen M, Kellosalo J. Immunonutrition in elective gastrointestinal surgery patients. *Scand J Surg.* 2007;96(1):46-50. doi: 10.1177/145749690709600109

12. Hübner M, Cerantola Y, Grass F, Bertrand PC, Schafer M, Demartines N. Preoperative immunonutrition in patients at nutritional risk: results of a double-blinded randomized clinical trial. *Eur J Clin Nutr.* 2012;66(7):850-855. doi: 10.1038/ejcn.2012.53

13. Hamilton-Reeves JM, Bechtel MD, Hand LK, et al. Effects of Immunonutrition for Cystectomy on Immune Response and Infection Rates: A Pilot Randomized Controlled Clinical Trial. *Eur Urol.* 2016;69(3):389-392. doi: 10.1016/j.eururo.2015.11.019

14. Mikagi K, Kawahara R, Kinoshita H, Aoyagi S. Effect of preoperative immunonutrition in patients undergoing hepatectomy; a randomized controlled trial. *Kurume Med J.* 2011;58(1):1-8. doi: 10.2739/kurumemedj.58.1

15. Okamoto Y, Okano K, Izuishi K, Usuki H, Wakabayashi H, Suzuki Y. Attenuation of the systemic inflammatory response and infectious complications after gastrectomy with preoperative oral arginine and omega-3 fatty acids supplemented immunonutrition. *World J Surg.* 2009;33(9):1815-1821. doi: 10.1007/s00268-009-0140-1

16. Seguin P, Locher C, Boudjema K, et al. Effect of a Perioperative Nutritional Supplementation with Oral Impact in Patients undergoing Hepatic Surgery for Liver Cancer: A Prospective, Placebo-Controlled, Randomized, Double-Blind Study. *Nutr Cancer.* 2016;68(3):464-472. doi: 10.1080/01635581.2016.1153670

17. Uno H, Furukawa K, Suzuki D, et al. Immunonutrition suppresses acute inflammatory responses through modulation of resolvin E1 in patients undergoing major hepatobiliary resection. *Surgery.* 2016;160(1):228-236. doi: 10.1016/j.surg.2016.01.019

18. Braga M, Gianotti L, Radaelli G, et al. Perioperative immunonutrition in patients undergoing cancer surgery: Results of a randomized double-blind phase 3 trial. *Arch Surg.* 1999;134(4):428-433. doi: 10.1001/archsurg.134.4.428

19. Braga M, Gianotti L, Nespoli L, Radaelli G, Di Carlo V. Nutritional approach in malnourished surgical patients: a prospective randomized study. *Arch Surg.* 2002;137(2):174-180. doi: 10.1001/archsurg.137.2.174

20. Braga M, Gianotti L, Vignali A, Carlo VD. Preoperative oral arginine and n-3 fatty acid supplementation improves the immunometabolic host response and outcome after colorectal resection for cancer. *Surgery.* 2002;132(5):805-814. doi: 10.1067/msy.2002.128350

21. Campillo M, Fernandez M, Salas AM, Rituerto C. A randomised controlled trial of preoperative oral immunonutrition in patients undergoing surgery for colorectal cancer: Hospital stay and health care costs. *Cir Cir.* 2016. doi: 10.1016/j.circen.2017.11.008

22. Felekis D, Eleftheriadou A, Papadakos G, et al. Effect of perioperative immuno-enhanced enteral nutrition on inflammatory response, nutritional status, and outcomes in head and neck cancer patients undergoing major surgery. *Nutr Cancer.* 2010;62(8):1105-1112. doi: 10.1080/01635581.2010.494336

23. Fujitani K, Tsujinaka T, Fujita J, et al. Prospective randomized trial of preoperative enteral immunonutrition followed by elective total gastrectomy for gastric cancer. *Br J Surg.* 2012;99(5):621-629. doi: 10.1002/bjs.8706

24. Gianotti L, Braga M, Nespoli L, Radaelli G, Beneduce A, Di Carlo V. A randomized controlled trial of preoperative oral supplementation with a specialized diet in patients with gastrointestinal cancer. *Gastroenterology.* 2002;122(7):1763-1770. doi: 10.1053/gast.2002.33587

25. Hamza N, Darwish A, O'Reilly DA, et al. Perioperative enteral immunonutrition modulates systemic and mucosal immunity and the inflammatory response in patients with periampullary cancer scheduled for pancreaticoduodenectomy: A randomized clinical trial. *Pancreas.* 2015;44(1):41-52. doi: 10.1097/MPA.0000000000000222

26. Horie H, Okada M, Kojima M, Nagai H. Favorable effects of preoperative enteral immunonutrition on a surgical site infection in patients with colorectal cancer without malnutrition. *Surg Today.* 2006;36(12):1063-1068. doi: 10.1007/s00595-006-3320-8

27. Kanekiyo S, Takeda S, Iida M, et al. Efficacy of perioperative immunonutrition in esophageal cancer patients undergoing esophagectomy. *Nutrition.* 2019;59:96-102. doi: 10.1016/j.nut.2018.08.006

28. McCarter MD, Gentilini OD, Gomez ME, Daly JM. Preoperative oral supplement with immunonutrients in cancer patients. *JPEN J Parenter Enteral Nutr.* 1998;22(4):206-211. doi: 10.1177/0148607198022004206

29. Moya P, Soriano-Irigaray L, Ramirez JM, et al. Perioperative Standard Oral Nutrition Supplements Versus Immunonutrition in Patients Undergoing Colorectal Resection in an Enhanced Recovery (ERAS) Protocol: A Multicenter Randomized Clinical Trial (SONVI Study). *Medicine.* 2016;95(21):e3704. doi: 10.1097/md.0000000000003704

30. Senkal M, Zumtobel V, Bauer KH, et al. Outcome and cost-effectiveness of perioperative enteral immunonutrition in patients undergoing elective upper gastrointestinal tract surgery: A prospective randomized study. *Arch Surg.* 1999;134(12):1309-1316. doi: 10.1001/archsurg.134.12.1309

31. Turnock A, Calder PC, West AL, Izzard M, Morton RP, Plank LD. Perioperative immunonutrition in well-nourished patients undergoing surgery for head and neck cancer: Evaluation of inflammatory and immunologic outcomes. *Nutrients.* 2013;5(4):1186-1199. doi: 10.3390/nu5041186
